# Supplementary material for: Cell-Size-Dependent Transcription of FLC and Its Antisense Long Non-coding RNA COOLAIR Explain Cell-to-Cell Expression Variation
Source: Cell Syst. 2017 Jun 28;4(6):622–635.e9. doi: 10.1016/j.cels.2017.05.010 (PMC5493185; doi:10.1016/j.cels.2017.05.010)
Supplement: Document S1. Figures S1–S4 [file mmc1.pdf]

Cell Systems, Volume *4*

Supplemental Information

Cell-Size-Dependent Transcription of *FLC* and Its Antisense Long Non-coding RNA *COOLAIR* Explain Cell-to-Cell Expression Variation

Robert Ietswaart, Stefanie Rosa, Zhe Wu, Caroline Dean, and Martin Howard

Supplemental Information

Figure legends

Figure S1. Related to Figure 2

Characterization of cellular *FLC* expression, mRNA degradation variation and cellular volume

(A) Fluorescence localization of *FLC* DNA as assayed by DNA-FISH, full length *FLC* intron 1 RNA as assayed by smFISH (red), and an overlay containing both the above and DAPI stain (blue) in representative ColFRI outer layer root cells. Scale bar: 5µm. Data previously published in (Rosa et al., 2016).

(B) Histogram of pooled cellular *FLC* mRNA distributions after treatment with DMSO (mock) for 4 h and 6 h (N=202 cells from 3 biological replicates).

(C) Histogram of cellular *FLC* mRNA counts after 6 h of ActD treatment (blue) and Poisson decay process prediction (red) with distribution of mRNA after 4 h ActD treatment (shown in Fig. 2E) as the initial distribution. Right panel: Cumulative distribution functions (CDFs) of the *FLC* mRNA count and model prediction.

(D) Scatter plot comparing two volume estimation methods: 3D segmentation vs projection method in ColFRI outer layer root cells from where *FLC* mRNA counts were recorded (N=209 cells from 8 biological replicates, Fig. 2C). Also shown is line with unit slope (black).

(**E**) Schematic of reactions implemented in stochastic Gillespie simulations of cellular *FLC* mRNA production, with either Poisson or ON/OFF dynamics, and degradation.

(F) Histogram of cell volume distribution (N=200 cells from 3 biological replicates) from root outer layer cells from where full length intron 1 foci counts were recorded (Fig. 3B,C). These cell volumes were used as an input into the stochastic simulations described in (E) to account for the variation in cell size affecting *FLC* transcription.

(**G**) Histograms of cellular mRNA model predictions from stochastic simulations described in (E) given the experimentally observed volume distribution described in (F). ON/OFF simulations had a burst size of 3 transcription events per ON/OFF cycle with either the burst frequency (k_{on}) or burst size (*bs*) scaling with cell volume. Right panel: cumulative distribution functions (CDFs) of *FLC* mRNA (from Fig. 2C) and model predictions.

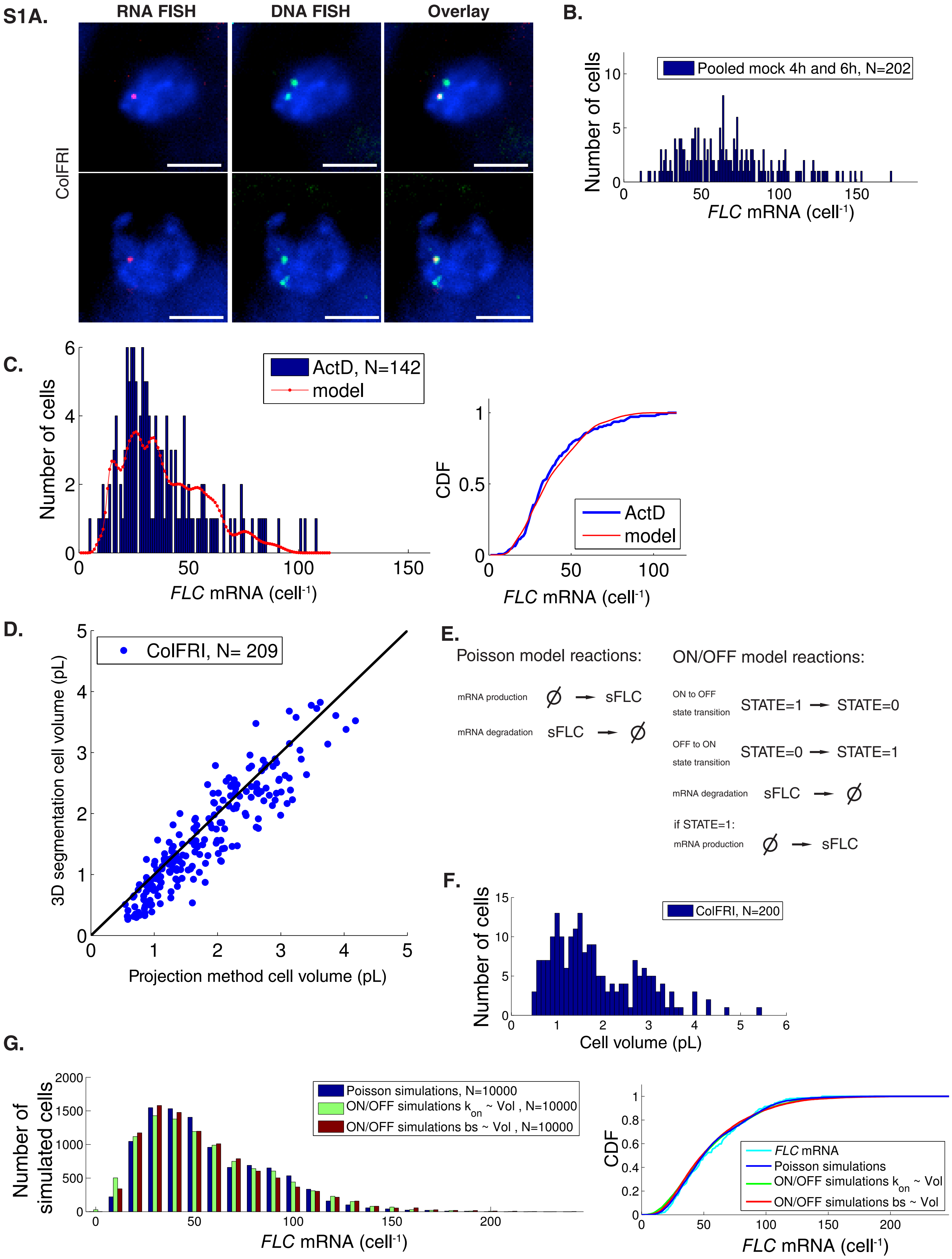
S1A.
RNA FISH
DNA FISH
Overlay
ColFRI
B.
Pooled mock 4h and 6h, N=202
Number of cells
FLC mRNA (cell-1)
C.
ActD, N=142
model
Number of cells
FLC mRNA (cell-1)
CDF
ActD
model
FLC mRNA (cell-1)
D.
ColFRI, N= 209
3D segmentation cell volume (pL)
Projection method cell volume (pL)
E.
Poisson model reactions:
mRNA production ∅ → sFLC
mRNA degradation sFLC → ∅
ON/OFF model reactions:
ON to OFF state transition STATE=1 → STATE=0
OFF to ON state transition STATE=0 → STATE=1
mRNA degradation sFLC → ∅
if STATE=1:
mRNA production ∅ → sFLC
F.
ColFRI, N=200
Number of cells
Cell volume (pL)
G.
Poisson simulations, N=10000
ON/OFF simulations kon ~ Vol , N=10000
ON/OFF simulations bs ~ Vol , N=10000
Number of simulated cells
FLC mRNA (cell-1)
CDF
FLC mRNA
Poisson simulations
ON/OFF simulations kon ~ Vol
ON/OFF simulations bs ~ Vol
FLC mRNA (cell-1)

Figure S2. Related to Figure 3

Estimation of Pol II elongation, *FLC* intron 1 processing and lariat degradation rates using a range of missing probe length fractions

(**A**) Marginal distributions for intron processing rate that generate good fits to the data shown in Fig. 3C according to a χ^2 test (degrees of freedom k=4, acceptance probability p≥0.1) with missing probe length fractions ranging from 0 to 1/3. Boxplots indicate: minimum, 25% quantile, median, 75% quantile and maximal values.

(**B**) As in (**A**) but for elongation rate.

(**C**) As in (**A**) but for 5' to 3' lariat degradation rate.

(**D**) As in (**A**) but for 3' to 5' lariat degradation rate.

(**E**) Volume dependence of replicates measuring cellular smFISH foci counts per cell in outer layer cells for four different *FLC* intron 1 probe sets: full length (full), middle (mid), 5' end and 3' end. Full length: two separate experiments were performed using respectively 4 and 3 biological replicates, respectively. Experiment 1: data as in Fig. 2B and 4C, experiment 2: data as in Fig. 3C. For the other probe sets (mid, 5' end and 3' end) replicates are shown as pooled data from two biological replicates each; for these, all biological replicates pooled together are shown in Fig. 3E,F. Error lines: s.e.m. as function of volume.

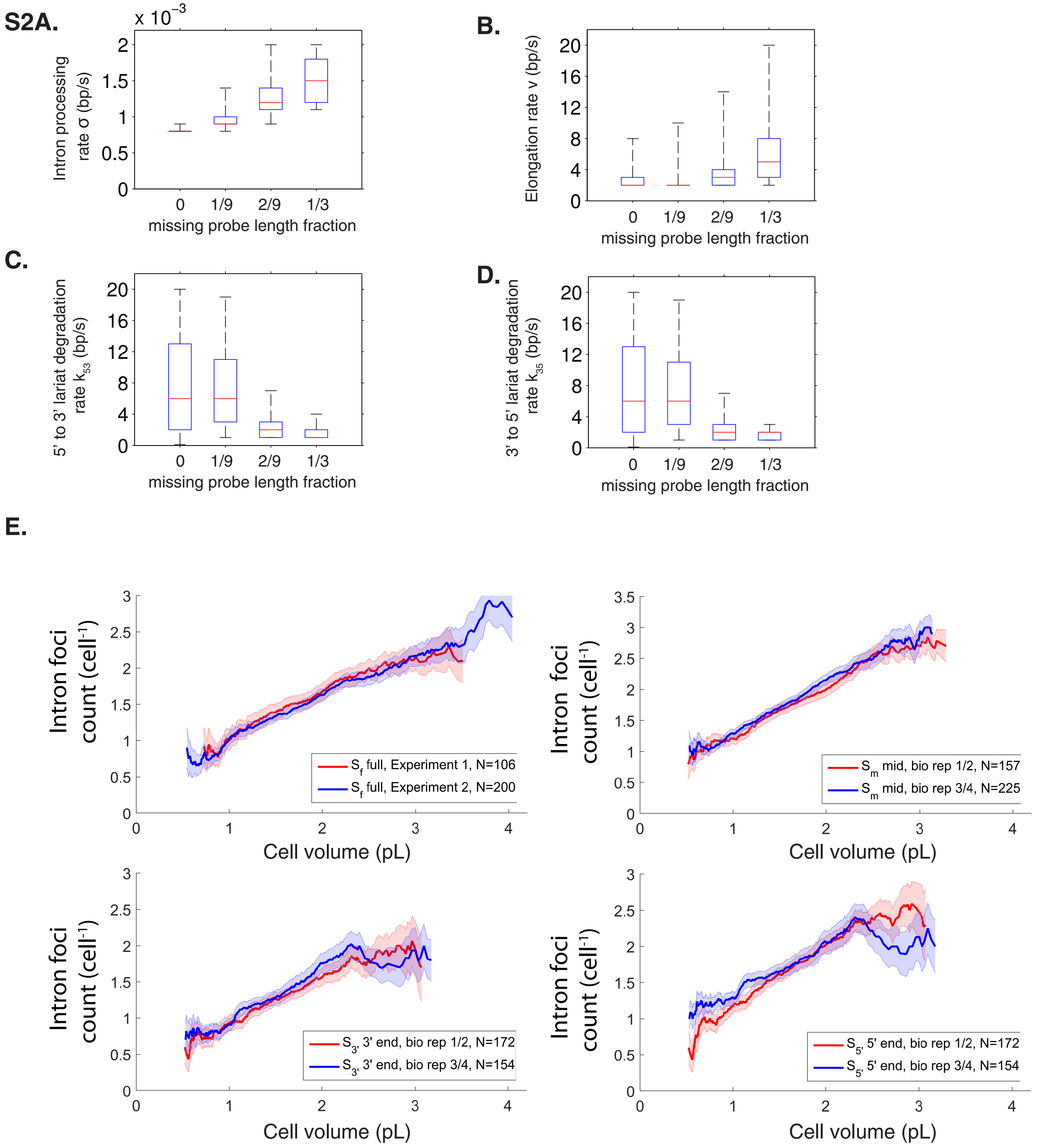
S2A.
x 10^-3
Intron processing rate σ (bp/s)
missing probe length fraction
0
1/9
2/9
1/3
B.
Elongation rate v (bp/s)
missing probe length fraction
C.
5' to 3' lariat degradation rate k53 (bp/s)
missing probe length fraction
D.
3' to 5' lariat degradation rate k35 (bp/s)
missing probe length fraction
E.
Intron foci count (cell-1)
Cell volume (pL)
Sf full, Experiment 1, N=106
Sf full, Experiment 2, N=200
Sm mid, bio rep 1/2, N=157
Sm mid, bio rep 3/4, N=225
S3' 3' end, bio rep 1/2, N=172
S3' 3' end, bio rep 3/4, N=154
S5' 5' end, bio rep 1/2, N=172
S5' 5' end, bio rep 3/4, N=154

Figure S3. Related to Figure 3

Spatiotemporal Gillespie simulations of *FLC* transcription to quantify *FLC* mRNA release from the locus

(A) Schematic of spatially discretized *FLC* gene and reactions as implemented in spatiotemporal stochastic Gillespie simulations of *FLC* transcription and RNA degradation dynamics. Sense (black) and antisense (grey) transcripts are indicated. Since antisense transcription is very low in root outer layer cells, we did not include antisense transcription in our simulations.

(B) Volume dependence of cellular smFISH foci counts per cell in outer layer cells for *FLC* intron 1 as in Fig 3E,F. In left panel: full length (full) and 5' end; in right panel: middle (mid) and 3' end. Also shown are spatiotemporal Gillespie simulation fits (black, grey). Error lines: s.e.m. as function of volume.

(**C**) Volume dependence of average locus associated exonic *FLC* RNA levels from two separate experiments (with 2 biological replicates per experiment). Three different quantification methods shown as in Fig. 3H, which shows the pooled data. Error lines: s.e.m. as a function of volume.

S3A.

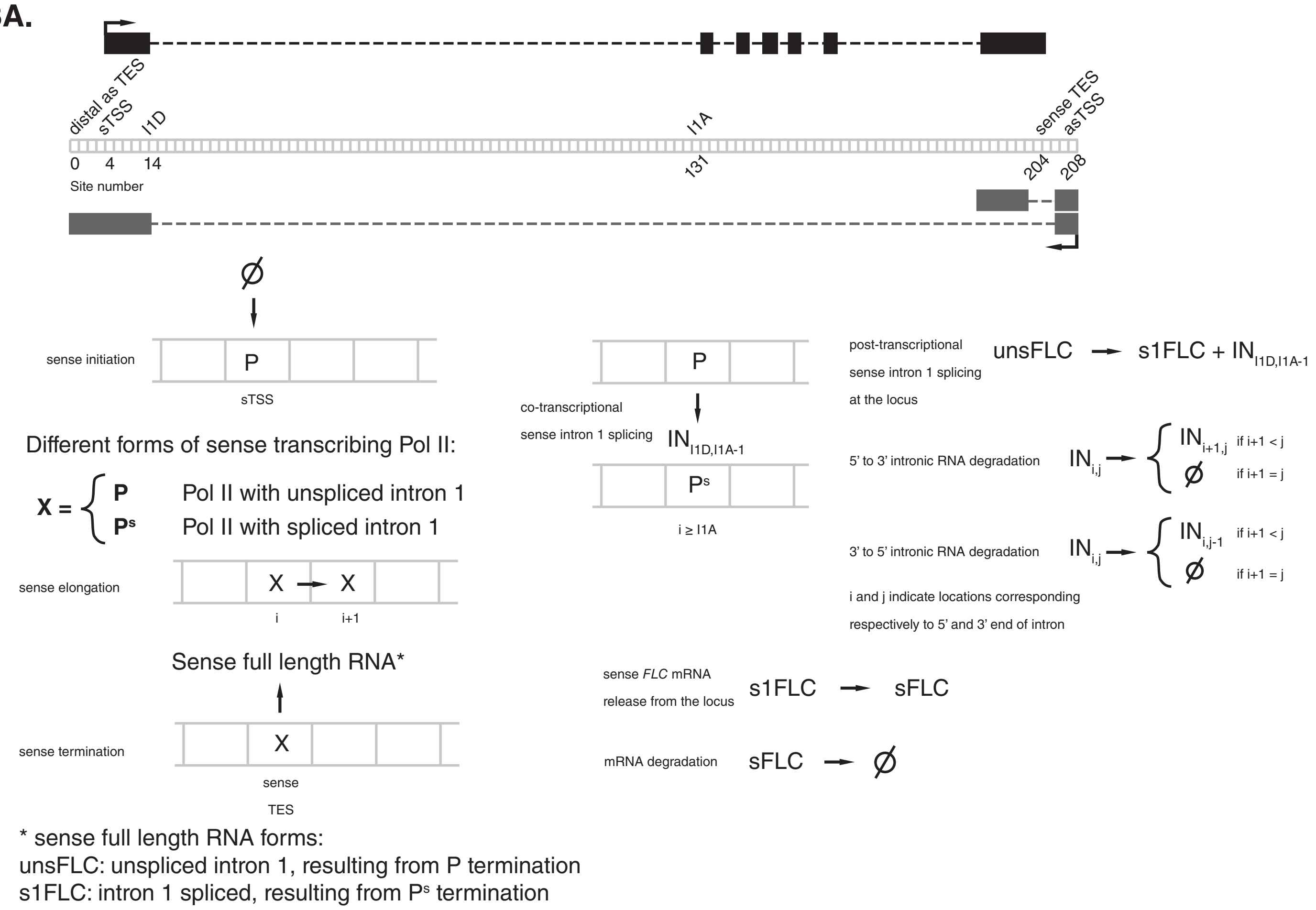
distal as TES
sTSS
I1D
I1A
sense TES
asTSS
0
4
14
131
204
208
Site number
sense initiation
P
sTSS
Different forms of sense transcribing Pol II:
X =
P Pol II with unspliced intron 1
Ps Pol II with spliced intron 1
sense elongation
X → X
i
i+1
Sense full length RNA*
sense termination
X
sense TES
* sense full length RNA forms:
unsFLC: unspliced intron 1, resulting from P termination
s1FLC: intron 1 spliced, resulting from Ps termination
co-transcriptional sense intron 1 splicing
P
IN I1D,I1A-1
Ps
i ≥ I1A
post-transcriptional sense intron 1 splicing at the locus
unsFLC → s1FLC + IN I1D,I1A-1
5' to 3' intronic RNA degradation
IN i,j → IN i+1,j if i+1 < j
Ø if i+1 = j
3' to 5' intronic RNA degradation
IN i,j → IN i,j-1 if i+1 < j
Ø if i+1 = j
i and j indicate locations corresponding respectively to 5' and 3' end of intron
sense FLC mRNA release from the locus
s1FLC → sFLC
mRNA degradation
sFLC → Ø

B.

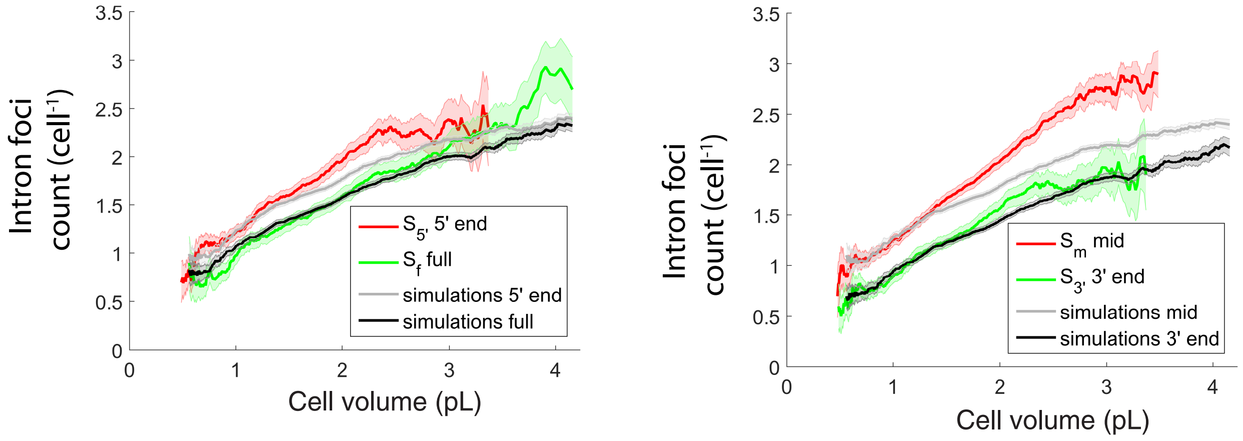
Intron foci count (cell⁻¹)
Cell volume (pL)
S5', 5' end
Sf full
simulations 5' end
simulations full
Sm mid
S3', 3' end
simulations mid
simulations 3' end

C.

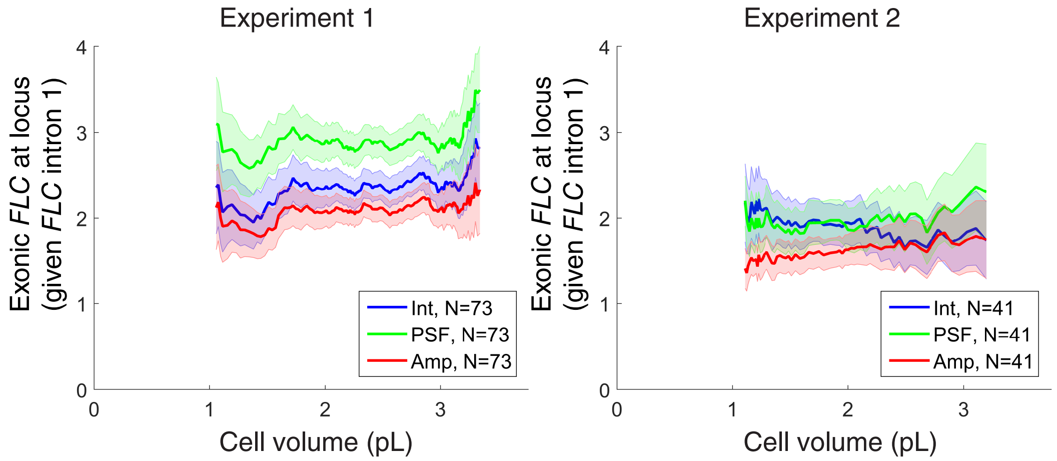
Experiment 1
Experiment 2
Exonic FLC at locus (given FLC intron 1)
Cell volume (pL)
Int, N=73
PSF, N=73
Amp, N=73
Int, N=41
PSF, N=41
Amp, N=41

Figure S4. Related to Figure 4.

Characterization of anticorrelation between *FLC* sense and antisense transcription

(A) Fluorescence localization of full length sense intron 1 (red) and antisense (*COOLAIR*) 5' end distal intron (green) overlaid with DAPI stain (blue) in representative ColFRI prevasculature root cell. Scale bar: 5µm. Data previously published in (Rosa et al., 2016).

(B) Frequency of loci containing *FLC* full length intron 1, when *COOLAIR* 5' end distal intron is not present (S) or present (S if AS) at the same locus in root prevasculature cells. N=254 cells from 6 biological replicates. Error bars: s.e.m. **** p-value < 10^{-4}. Data previously published in (Rosa et al., 2016).

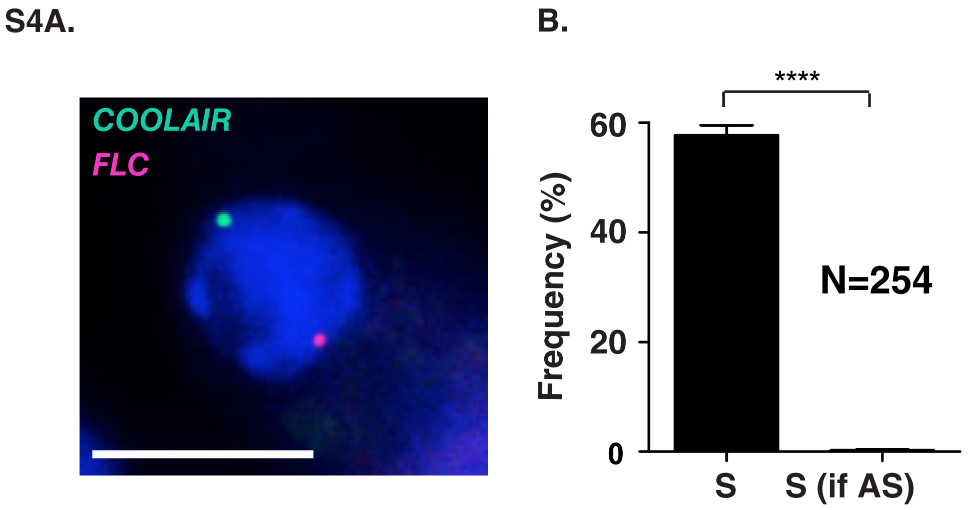
S4A.
COOLAIR
FLC
B.
****
Frequency (%)
60
40
20
0
N=254
S
S (if AS)
