# Supplementary material for: Cell-Size-Dependent Transcription of FLC and Its Antisense Long Non-coding RNA COOLAIR Explain Cell-to-Cell Expression Variation
Source: Cell Syst. 2017 Jun 28;4(6):622–635.e9. doi: 10.1016/j.cels.2017.05.010 (PMC5493185; doi:10.1016/j.cels.2017.05.010)
Supplement: Document S2. Article plus Supplemental Information [file mmc3.pdf]

Cell Systems

Article

Cell-Size-Dependent Transcription of *FLC* and Its Antisense Long Non-coding RNA *COOLAIR* Explain Cell-to-Cell Expression Variation

Graphical Abstract

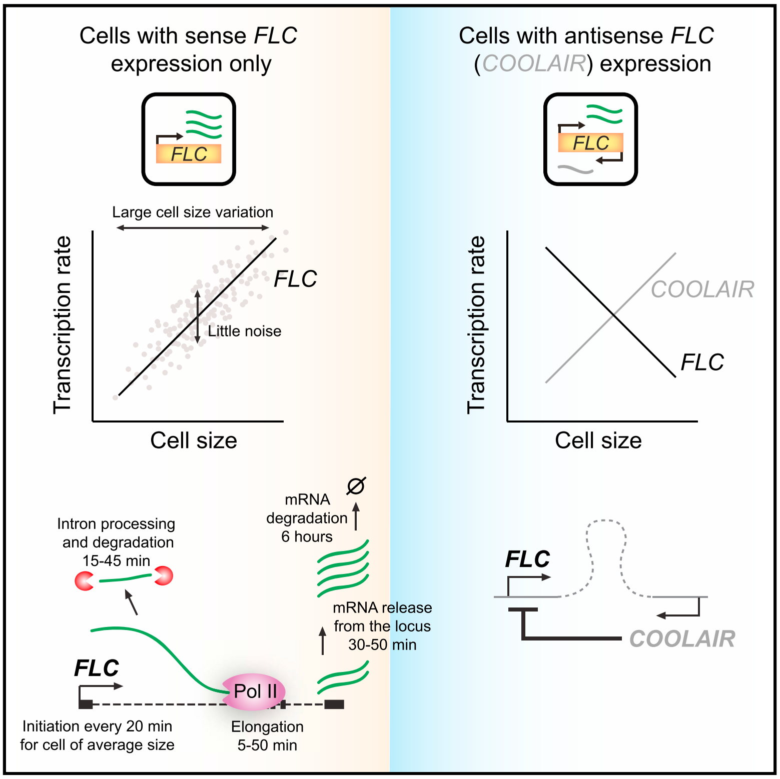

Highlights

- The kinetics and variability of *FLC* expression are quantified in single cells
- Without antisense transcription, *FLC* mRNA production scales with cell size
- *FLC* transcription shows minimal stochasticity and no large bursts
- In cells transcribing the antisense RNA *COOLAIR*, size scaling of *FLC* is altered

Authors

Robert Ietswaart, Stefanie Rosa, Zhe Wu, Caroline Dean, Martin Howard

Correspondence

caroline.dean@jic.ac.uk (C.D.),
martin.howard@jic.ac.uk (M.H.)

In Brief

Ietswaart et al. quantify the kinetics of *Arabidopsis FLOWERING LOCUS C* (*FLC*) expression on the single-cell level. They reveal that while *FLC* expression is variable from cell to cell, this is not due to noise. Instead, variability arises from a linear scaling between *FLC* mRNA number and cell size. The size scaling of *FLC* expression can be modulated by transcription of its antisense long non-coding RNA, *COOLAIR.*

Ietswaart et al., 2017, Cell Systems *4*, 622–635
June 28, 2017 © 2017 The Author(s). Published by Elsevier Inc.
http://dx.doi.org/10.1016/j.cels.2017.05.010

OPEN ACCESS
CellPress

Cell Systems
Article

Cell-Size-Dependent Transcription of *FLC* and Its Antisense Long Non-coding RNA *COOLAIR* Explain Cell-to-Cell Expression Variation

Robert Ietswaart,[1,2,3,6] Stefanie Rosa,[2,4,6] Zhe Wu,[2,5,6] Caroline Dean,[2,*] and Martin Howard[1,2,7,*]
[1]Computational and Systems Biology
[2]Department of Cell and Developmental Biology
John Innes Centre, Norwich Research Park, Norwich NR4 7UH, UK
[3]Present address: Department of Genetics, Harvard Medical School, Boston, MA 02115, USA
[4]Present address: Institute of Biochemistry and Biology, Plant Physiology, University of Potsdam, 14476 Potsdam-Golm, Germany
[5]Present address: Department of Biology, Southern University of Science and Technology, Shenzhen 518055, China
[6]These authors contributed equally
[7]Lead Contact
*Correspondence: caroline.dean@jic.ac.uk (C.D.), martin.howard@jic.ac.uk (M.H.)
http://dx.doi.org/10.1016/j.cels.2017.05.010

SUMMARY

Single-cell quantification of transcription kinetics and variability promotes a mechanistic understanding of gene regulation. Here, using single-molecule RNA fluorescence in situ hybridization and mathematical modeling, we dissect cellular RNA dynamics for *Arabidopsis FLOWERING LOCUS C* (*FLC*). *FLC* expression quantitatively determines flowering time and is regulated by antisense (*COOLAIR*) transcription. In cells without observable *COOLAIR* expression, we quantify *FLC* transcription initiation, elongation, intron processing, and lariat degradation, as well as mRNA release from the locus and degradation. In these heterogeneously sized cells, *FLC* mRNA number increases linearly with cell size, resulting in a large cell-to-cell variability in transcript level. This variation is accounted for by cell-size-dependent, Poissonian *FLC* mRNA production, but not by large transcriptional bursts. In *COOLAIR*-expressing cells, however, antisense transcription increases with cell size and contributes to *FLC* transcription decreasing with cell size. Our analysis therefore reveals an unexpected role for antisense transcription in modulating the scaling of transcription with cell size.

INTRODUCTION

A thorough understanding of gene regulation requires an accurate quantification of the kinetic parameters influencing the transcription cycle. The rates of transcript production and mRNA degradation directly determine mRNA concentrations (Bentley, 2014; Dolken et al., 2008; Padovan-Merhar et al., 2015; Sidaway-Lee et al., 2014; Wu et al., 2016). However, transcription elongation, intron splicing, and mRNA release from the locus can also feed through to modulate mRNA levels (Bentley, 2014; de la Mata et al., 2003; Hazelbaker et al., 2013; Stuparevic et al., 2013), for example where kinetic coupling occurs (Bentley, 2014; de la Mata et al., 2003; Hazelbaker et al., 2013). Such kinetic couplings between RNA polymerase II (Pol II) elongation, splicing, or termination can quantitatively control the formation of alternative mRNA isoforms (Bentley, 2014).

An element that can affect precise control of gene expression is stochasticity (Chubb and Liverpool, 2010; Corrigan et al., 2016; Dar et al., 2012; Elowitz et al., 2002; Huh and Paulsson, 2011; Lenstra et al., 2015; Raj et al., 2006, 2010; Raj and van Oudenaarden, 2008; Shahrezaei and Swain, 2008; Sherman et al., 2015; Skinner et al., 2016; Thattai, 2016; Zenklusen et al., 2008; Zopf et al., 2013). Any comprehensive quantification of RNA dynamics must therefore also take into account its degree of variability. Fundamentally, transcription and RNA degradation are both stochastic processes. How transcriptional output could be influenced by intrinsic stochasticity has been intensively studied (Chubb and Liverpool, 2010; Corrigan et al., 2016; Dar et al., 2012; Elowitz et al., 2002; Huh and Paulsson, 2011; Lenstra et al., 2015; Raj et al., 2006, 2010; Raj and van Oudenaarden, 2008; Shahrezaei and Swain, 2008; Skinner et al., 2016; Thattai, 2016; Zenklusen et al., 2008). Nevertheless, it still remains unclear as to what extent variation previously often attributed to intrinsic stochasticity is actually caused by extrinsic variation due to cell cycle, cell size, or other fundamentally deterministic features (Battich et al., 2015; Kempe et al., 2015; Padovan-Merhar et al., 2015; Sherman et al., 2015; Zopf et al., 2013).

Measuring the kinetics and variability of transcription and RNA processing in vivo is a challenging task in multicellular organisms. Previous approaches have focused on parts of the RNA life cycle (e.g., production/degradation), or have provided only relative, not absolute kinetic measurements (Battich et al., 2015; Corrigan et al., 2016; Dolken et al., 2008; Elowitz et al., 2002; Huh and Paulsson, 2011; Padovan-Merhar et al., 2015; Raj et al., 2006; Sidaway-Lee et al., 2014; Skinner et al., 2016; Thattai, 2016; Wu et al., 2016). In this study, we combine single-cell assays with mathematical modeling to comprehensively quantitate in vivo the transcription and RNA dynamics of

This is an open access article under the CC BY license (http://creativecommons.org/licenses/by/4.0/).

OPEN
ACCESS
CellPress

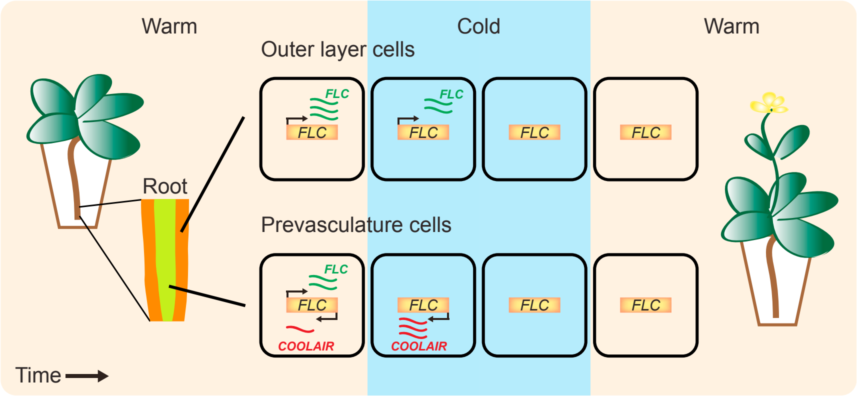

Figure 1. Overview of Transcriptional Regulation at *FLC*

Schematic indicating the regulation of transcription at *FLC*, which encodes a MADS-box transcription factor that represses the *Arabidopsis* transition to flowering (Michaels and Amasino, 1999; Sheldon et al., 1999). The *FLC* locus is also transcribed from its 3′ end in the antisense direction, resulting in a group of long non-coding transcript isoforms collectively termed *COOLAIR* (Swiezewski et al., 2009). In warm conditions, as investigated in this study, unspliced *COOLAIR* is expressed in root prevasculature cells (inner layer tissue) but was not detected in outer layer cells (mostly from the epidermis and cortex) (Rosa et al., 2016). *FLC* levels are quantitatively controlled through the antagonistic *FRI* and Autonomous pathways (Crevillén and Dean, 2011; Ietswaart et al., 2012), which respectively activate and repress *FLC* transcription. A short period of winter cold temperatures leads to a transient induction of *COOLAIR* transcription and concomitant *FLC* transcriptional repression (Rosa et al., 2016; Swiezewski et al., 2009). Prolonged winter cold induces epigenetically stable repression of the *FLC* locus through the process of vernalization (Angel et al., 2011; Berry and Dean, 2015; Song et al., 2012).

FLOWERING LOCUS C (*FLC*) (Michaels and Amasino, 1999; Sheldon et al., 1999), a key quantitative developmental regulator in *Arabidopsis thaliana* (Figure 1). *FLC* encodes a MADS-box transcription factor that functions as a repressor of the transition to flowering. In warm conditions, *FLC* is regulated by two antagonistic pathways: it is upregulated through the transcriptional activator *FRIGIDA* (*FRI*) (Crevillén and Dean, 2011), and repressed by the so-called Autonomous pathway (Ietswaart et al., 2012). The latter is mediated in part by a group of antisense long non-coding transcripts, termed *COOLAIR* (Figure 1), whose transcription start site is located immediately downstream of the *FLC* poly(A) site (Swiezewski et al., 2009). *COOLAIR* expression is tissue specific and in warm conditions *COOLAIR* is observed in root prevasculature cells (Figure 1) (Rosa et al., 2016). The quantitative level of *FLC* established by these antagonistic Autonomous and *FRI* pathways determines whether the plant goes through winter before flowering. If such overwintering does occur, cellular *FLC* expression is epigenetically silenced by the prolonged cold of winter, through the process of vernalization (Figure 1) (Berry and Dean, 2015). Such silencing is a stochastic all-or-nothing effect at individual *FLC* loci, but where the fraction of silenced loci increases quantitatively with an increasing duration of cold exposure (Angel et al., 2011; Song et al., 2012). However, how quantitative regulation and stochasticity interplay to determine *FLC* expression in warm conditions has remained unclear.

In this work, we determine the kinetics of *FLC* mRNA production and degradation, Pol II elongation, intron processing, lariat degradation, and mRNA release in cells without observable *COOLAIR* expression. We also quantitate the degree of stochasticity in the dynamics of *FLC* RNA. We observe large cell-to-cell variability in *FLC* mRNA numbers but find that it is not due to intrinsic stochasticity. Instead, it is well explained by a linear scaling of transcript number with cell size. We show that this size scaling results from a total cellular mRNA production that increases linearly with cell size. Our findings are consistent with the entire *FLC* RNA dynamics being minimally stochastic. Finally, in *COOLAIR*-expressing cells it is antisense transcription that scales with cell size, which contributes to *FLC* transcription now decreasing with cell size. Our work therefore reveals an unexpected role for antisense transcription in modulating the cell size dependence of sense transcription.

RESULTS

Cell-to-Cell Variability of *FLC* mRNA Is Larger than Predicted from Poisson Production and Degradation Processes

We utilized single-molecule fluorescence in situ hybridization (smFISH) (Duncan et al., 2016; Raj et al., 2008; Rosa et al., 2016) in the *A. thaliana* Columbia ecotype with an active *FRI* allele (ColFRI) to measure single-cell RNA levels in warm conditions. The samples were prepared using a root squash method that typically yields single cell layers that originate from the outer cell layers of the root (mostly from the epidermis and cortex) (Figure 1), with cells that do not express observable *COOLAIR* (Rosa et al., 2016). DAPI stain was then used to label nuclei and two distinct smFISH probe sets employed to visualize *FLC* RNA: one covering sense *FLC* exons (*FLC* mRNA) and the second covering sense intron 1 (Figure 2A). Intron 1 *FLC* signal was only detected in the nucleus (Figure 2A). Using consecutive smFISH and DNA FISH, we found that intron 1 *FLC* co-localized exclusively with *FLC* loci (Figure S1A), indicating that sense intron 1 splicing and lariat degradation occurs at the locus. This finding therefore enabled us to use the *FLC* intron 1 signal to label transcriptionally active *FLC* loci. Intron 1 *FLC* foci were found with counts ranging from 0 up to 4 per cell, with most cells exhibiting at least one *FLC* intron 1 focus (Figure 2B). In *Arabidopsis* sister-chromatid cohesion is variable and incomplete (Schubert et al., 2006), meaning that the presence of a maximum of four intronic foci per cell is consistent with these root cells being diploid (Hayashi et al., 2013; Yin et al., 2014). Below, we quantify the underlying transcription and RNA degradation kinetics responsible for these observations.

Exonic signal was abundant and mostly cytoplasmic, indicating that *FLC* mRNA can be easily detected with the appropriate probe set (Figure 2A). The cellular distribution of *FLC* mRNA counts was unimodal with a mean of 58 ± 2 molecules (Figure 2C). Here and elsewhere, unless stated otherwise, we report the mean (±SEM). We estimate our mRNA counting error

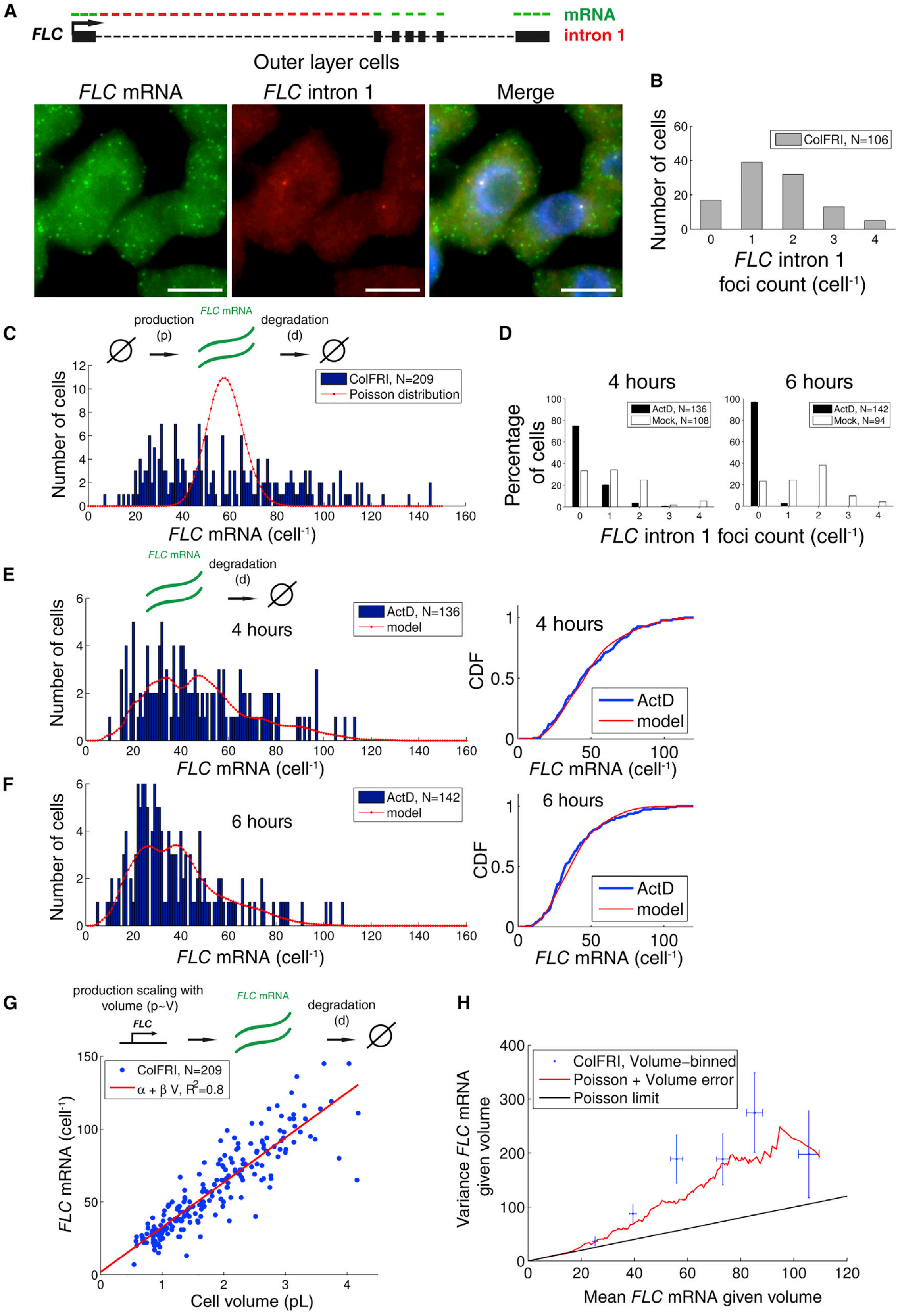

(legend on next page)

to be at most 4% by comparing our algorithm (Duncan et al., 2016; Olsson and Hartley, 2016) with FISH-quant, a separate counting method (Mueller et al., 2013). By comparing these cellular levels with a Poisson distribution (Figure 2C), we found that the experimental mRNA distribution from single cells is much broader than for the Poisson case (Kolmogorov-Smirnov [KS] test, $p = 10^{-25}$): the distribution variance is ~14-fold larger than the mean. Assuming that cellular mRNA production and degradation occur as Poisson processes, i.e., independently of each other and with constant probabilities p and d per unit time, respectively (Chubb and Liverpool, 2010; Gardiner, 2009; Shahrezaei and Swain, 2008), we would expect a Poisson distribution for such a birth-death process (Chubb and Liverpool, 2010; Gardiner, 2009; Shahrezaei and Swain, 2008; Thattai, 2016), with mean mRNA copy number p/d. We therefore conclude that the cellular *FLC* mRNA variation cannot be explained by a birth-death process with constant production and degradation probabilities per unit time.

FLC mRNA Degradation Is Well Described by a Poisson Process with a Constant Half-Life

To determine whether *FLC* mRNA degradation might be responsible for the observed broad cell-to-cell variation in mRNA levels, we considered what would happen to the *FLC* mRNA distribution after inhibition of transcription. Initially the mRNA distribution would be as observed in Figure 2C, but as time progresses mRNA levels will decrease. A simple hypothesis is that mRNA degradation occurs with a constant degradation rate d (units: s^{-1}). The degradation rate d can then be estimated by fitting an exponential function $R(t) = R(0)\,exp\,(-d\,t)$ to the experimentally measured mean mRNA levels (Dolken et al., 2008; Gardiner, 2009; Sidaway-Lee et al., 2014). The corresponding Poisson stochastic process is characterized by a constant degradation probability per unit time d (Gardiner, 2009). If mRNA degradation is well described by this Poisson model, which exhibits inherently minimal stochasticity, the cellular mRNA distribution after transcription inhibition should be completely determined by the mRNA distribution before inhibition and the degradation probability per unit time d. To probe this hypothesis, we first derived the probability distribution for the number of mRNA molecules, r, as a function of time $P(r,t)$, given the observed initial distribution $P(r,0)$ before transcriptional inhibition (STAR Methods). This derivation resulted in:

$$P(r,t) = \sum_{b=r}^{M} \binom{b}{r} (\exp(-dt))^{r} (1-\exp(-dt))^{b-r} P(b,0) \qquad \text{(Equation 1)}$$

where M indicates the maximum amount of cellular mRNA observed in the initial distribution.

To experimentally test whether *FLC* mRNA degradation is well described by this model with minimal associated stochasticity, we treated plant seedlings with the transcription elongation inhibitor actinomycin D (ActD) (Dolken et al., 2008). We then subsequently performed smFISH in a time series. We observed that intron 1 *FLC* foci were almost completely absent as compared with a DMSO-treated sample (mock) after 4 hr and 6 hr of ActD addition, indicating that transcription was indeed inhibited (Figure 2D). We used the experimentally measured mean mRNA count levels $\langle R_{\exp}(t)\rangle$ after 4 and 6 hr of treatment to estimate the mean degradation rate d: $\frac{\langle R_{\exp}(4\ hr)\rangle}{\langle R_{\exp}(6\ hr)\rangle} = \exp[d(6\ hr - 4\ hr)]$. This resulted in $d = 3.3 \pm 0.1 \times 10^{-5}\,s^{-1}$, equivalent to a half-life of approximately $t_{1/2} \approx 6\,hr$.

Using the estimated degradation rate, we then compared the stochastic model prediction from Equation 1 with the experimentally observed mRNA count distributions after 4 hr and 6 hr (Figures 2E and 2F). Here, we used the pooled mock treated *FLC* mRNA count distributions (Figure S1B) as the initial distribution in the model to minimize the influence of cell size variation (see below) between experiments. Furthermore, we assumed that mRNA degradation started after a time lag τ of 1 hr after ActD treatment (through replacing t by $t - \tau$ in Equation 1 for t = 4 or 6 hr). Such a time lag is reasonable considering the ActD penetration time into the plant tissue. The cumulative distribution functions of the model and experiments were then indeed similar at both the 4- and 6-hr time points, as shown in Figures 2E and 2F (KS test, p = 0.66 and 0.21 for 4 and 6 hr, respectively). Furthermore, starting from the experimental 4-hr distribution as an initial condition, we could correctly predict the entire 6-hr distribution using the same degradation rate but with no time lag in Equation 1 (KS test, p = 0.48) (Figure S1C). Note that, since our

Figure 2. Cellular Variation of *FLC* mRNA Production and Degradation in Outer Layer Root Cells

(A) Schematic of *FLC* gene with exons (boxes) and introns (dashed lines) indicated, and different probe sets for labeling *FLC* mRNA (green) and *FLC* full-length intron 1 (red). Fluorescence localization (z-stack projection) of *FLC* mRNA, full-length intron 1, and an overlay containing mRNA, intron 1 *FLC*, and DAPI stain (blue) in representative ColFRI outer layer root cells. Scale bar, 5 μm.

(B) Cellular *FLC* intron 1 foci counts (gray, n = 106 cells from four biological replicates, i.e., roots from different plants).

(C) Histogram of cellular mRNA foci counts (blue, n = 209 cells from eight biological replicates) and a Poisson distribution fit (red), which would be expected from a simple birth/death process of *FLC* mRNA production/degradation with constant probabilities per unit time (see schematic above).

(D) Cellular *FLC* intron 1 foci counts after treatment with ActD or DMSO (mock) for 4 hr and 6 hr. With ActD, after 4 hr and 6 hr the vast majority of intron 1 signal has disappeared. Mock-treated *FLC* mRNA distributions are shown in Figure S1B.

(E) Left panel: histogram of cellular *FLC* mRNA foci counts after 4 hr of ActD treatment (blue) and Poisson decay process prediction (red). Right panel: cumulative distribution functions (CDFs) of the *FLC* mRNA foci counts and Poisson decay process prediction. Schematic above histogram shows *FLC* mRNA degradation only, as appropriate for ActD treatment.

(F) As in (E) but after 6 hr of ActD treatment. Cells in (D), (E), and (F) are pooled from three biological replicates for each treatment and time point.

(G) Scatterplot of cellular *FLC* mRNA foci counts (same data as in C; blue) as a function of cell volume, together with least-squares fit (red). Schematic above shows *FLC* mRNA production increasing with cell volume and volume-independent degradation.

(H) Variance plotted against mean of cellular *FLC* mRNA foci counts, both for given binned cell volume (ColFRI, Volume-binned). Also shown is the behavior predicted by model with Poisson probability distribution with parameter βV(Poisson), as well as a Poisson model that takes into account volume measurement error (Poisson + Volume error). All error bars indicate standard errors on the respective quantities.

time window of 2 hr is relatively short compared with the cell cycle time (17 hr) for these *Arabidopsis* meristematic root cells (Hayashi et al., 2013; Yin et al., 2014), a reduction in mRNA levels due to dilution during cell division (Huh and Paulsson, 2011) is unlikely to affect these results. We conclude that *FLC* mRNA degradation is well described by a Poisson process with a constant half-life of ~6 hr in all outer layer cells. The observed broad distribution (Figure 2C) is therefore not caused by variation in *FLC* mRNA degradation.

Cellular *FLC* mRNA Levels Scale Linearly with Cell Size, Generated by a Similar Scaling of the Total mRNA Production Rate

Visual inspection suggested that cellular *FLC* mRNA levels could increase with cell size as observed for certain genes in mammalian cells (Kempe et al., 2015; Padovan-Merhar et al., 2015). We therefore asked whether the broad distribution of *FLC* mRNA counts could be influenced by cell-to-cell variability in cell size. We quantified cellular volume using two separate methods (STAR Methods), which gave consistent measures to within ≈20% (Figure S1D). We then compared cell volume with the corresponding cellular mRNA counts (Figure 2G). We found a strong linear correlation between *FLC* mRNA (R) and cell volume (V) using linear regression: $R = \alpha + \beta V$ (R^2-statistic = 0.8, F-statistic: p = 3×10^{-74}), with a slope $\beta = 31 \pm 1$ pL^{-1} (Figure 2G). The intercept α was not significantly different from zero (p = 0.4). Given that *FLC* degradation is well described by a Poisson process with a cell size-independent degradation probability per unit time, we conclude that the total cellular *FLC* mRNA production rate would need to increase linearly with volume, $p(V) = \beta V d$, in order to explain the observed linear scaling between mRNA and cell volume.

The *FLC* mRNA Distribution Is Consistent with Minimally Stochastic Poisson Dynamics

To disentangle how much of the cell-to-cell variation in *FLC* mRNA is governed by cell size under warm conditions, we can consider the cellular mRNA levels as a random variable such that its mean value as a function of cell volume V is given by the observed linear relationship: $R(V) = \beta V$ (Padovan-Merhar et al., 2015). Here, β is the slope of the fit in Figure 2G. If the major determinant of the variation would be cell volume with minimal residual variation, then the resulting distribution for a given volume would be Poisson, characterized by a mean and variance being both equal to βV (Figure 2H, Poisson). To test this hypothesis, we binned the experimental *FLC* mRNA levels according to volume (bin width: 0.5 pL) and computed the mean and variance for the experimental binned data (Figure 2H, experiments). Indeed, in cells with larger sizes, the mean and variance of *FLC* mRNA levels increased. However, the variances were systematically higher than the Poisson limit (Figure 2H). The above analysis assumes cell volumes are known precisely. In practice, our measurement error of ≈20% for estimating cell volumes (Figure S1D) generates additional uncertainty in the relation between cellular volume and mRNA levels. This uncertainty feeds through into a lower limit on our observable variance (for a given cell volume) that is higher than the Poisson limit (see derivation in STAR Methods). When we also took into account our error in volume estimation, we found that our experimental data are in fact well described by an underlying Poisson distribution (Figure 2H, Poisson + Volume error). To further establish that our residual variation is consistent with a Poisson distribution, we calculated the volume-corrected noise measure $N_R = \frac{Var(R_{\exp})}{\langle R_{\exp} \rangle^2} - \frac{Cov(R_{\exp}, V_{\exp})}{\langle R_{\exp} \rangle \langle V_{\exp} \rangle}$ (STAR Methods and Padovan-Merhar et al., 2015), where $V_{\exp}$ are the experimentally measured cell volumes. This expression for N_R can be evaluated directly from our data, resulting in $N_R = 0.04 \pm 0.01$, which is indeed close to the Poisson limit $\frac{1}{\langle R_{\exp} \rangle} = 0.02$. For comparison, N_R has been found to reach values above 1 for certain mammalian high-noise genes (Padovan-Merhar et al., 2015). Altogether, our analysis shows that cell size is the major source of cell-to-cell variation in *FLC* mRNA levels. Moreover, after controlling for volume measurement errors, the observed residual variation is consistent with minimal, Poisson variation.

To further test that our mRNA distribution can be explained by cell size variation and Poisson residual variation, we performed stochastic simulations using a Gillespie algorithm (Slepoy et al., 2008) of cellular mRNA production and degradation (Figure S1E and STAR Methods). Degradation was simulated with a constant degradation probability per unit time as described above. The total cellular mRNA production probability per unit time was given by $p(V) = \beta V d$. To approximate the deterministic cell-to-cell variation in mRNA production, we used our experimentally observed cell size distribution as an input (Figure S1F). As expected, with this procedure we could explain the cellular mRNA distribution (Figure S1G, KS test: p = 0.35), as well as its variance (Levene's test: p = 0.53).

The *FLC* mRNA Distribution Is Inconsistent with Large Transcriptional Bursts

To discern whether alternative mechanisms could also explain the mRNA distribution, we altered the above cell-size-dependent Poisson model to include an OFF state in which the production rate was zero, but which could switch back and forth to an ON state characterized by an mRNA production probability per unit time p_{on} (Figure S1E and STAR Methods). This ON/OFF model can exhibit "bursty" transcription kinetics, whereby multiple transcripts are being produced in bursts as opposed to uncorrelated single transcription events generated by a Poisson process. The transition probability per unit time from OFF to ON is termed the burst frequency (k_{on}), whilst the mean number of transcripts produced per ON-OFF cycle is termed the burst size $bs = \frac{p_{on}}{k_{off}}$ (Padovan-Merhar et al., 2015). Here, k_{off} indicates the transition probability per unit time from the ON to the OFF state. Transcription occurs in bursts when $k_{off} \gg k_{on}$ with a burst size $bs \gg 1$ (Dar et al., 2012; Shahrezaei and Swain, 2008). We fixed $k_{off} = 0.1\ s^{-1}$, which effectively ensured that the first condition was met in our simulations, then systematically increased the burst size from 1 upward. The average production rate as determined above is approximately the product of burst size and burst frequency: $p(V) = \beta V d = \frac{p_{on}}{k_{off}} \frac{1}{1 + \frac{k_{on}}{k_{off}}} k_{on} \cong bs\ k_{on}$. We then chose to allow either burst frequency or burst size scale

with volume, the latter as proposed for mammalian genes (Padovan-Merhar et al., 2015). In this way the remaining a priori unknown parameters k_{on} and p_{on} were also specified (STAR Methods). We found that the ON/OFF model was only consistent with our cellular *FLC* mRNA distribution if the burst size was maximally three transcripts for both the cases where burst size or burst frequency scale with volume (Figure S1G, Levine's [variance] test: p = 0.10 and 0.05, respectively; see STAR Methods for additional statistical test results). For a volume-dependent burst size, this number reflects the burst size for a cell of average volume (V = 1.8 pL). Increasing the burst size further led to too broad an *FLC* mRNA distribution (Levine's test: $p < 0.05$), due to the considerable fraction of cells in either the transcriptionally inactive or "bursty" states. Burst sizes compatible with our data (at most three transcripts) do not reflect a very "bursty" transcriptional mode, and are rather similar to Poisson transcription which can be interpreted in terms of our ON/OFF model as having a burst size per locus of 1. Altogether we conclude that, for a given volume, *FLC* transcriptional dynamics are inconsistent with large transcriptional bursts.

Estimation of *FLC* Transcription Initiation Rates

Since we have quantified cellular mRNA levels, we can utilize these data to infer the mean absolute transcript production rate per locus F in root outer layer cells. This quantification of F is important for a full quantitative understanding of *FLC* transcription, and will also be necessary to subsequently quantify further RNA dynamics, such as intron processing. The average cellular mRNA levels are the ratio of production and degradation: $\langle R \rangle = \frac{p}{d} = \frac{N_{loci}F}{d}$, where N_{loci} is the number of *FLC* gene copies. Although the mean degradation rate is constant during the cell cycle, the number of loci and potentially also the production rate per locus will vary. However, averaging mRNA levels over all observed cells is equivalent to averaging over the cell cycle because in this tissue, cells cycle constantly and asynchronously. We can thus consider an average copy number N_{loci} = 2.5 and mean mRNA production rate per locus F arising from time averaging over the *Arabidopsis* root meristematic cell cycle time scales (STAR Methods). Above we both experimentally measured $\langle R \rangle$ and determined the mean *FLC* mRNA degradation rate $d = 3.3 \pm 0.1 \times 10^{-5}\ s^{-1}$. From the above formula for $\langle R \rangle$, we can therefore extract the mean production rate per locus of $F = 7.5 \pm 0.4 \times 10^{-4}\ s^{-1}$, approximately once per 20 min. This estimate should be interpreted as an average over the cell cycle and relevant to a cell of average volume 1.8 pL. Altering the relevant cell cycle time scales by up to an hour resulted in N_{loci} ranging from 2.4 to 2.8 (STAR Methods). It is also possible that the production rate per locus could dynamically change during the cell cycle to buffer against changes in gene copy number, as found for mammalian genes (Padovan-Merhar et al., 2015). This case leads to N_{loci} = 2.7 (STAR Methods). In all of these cases, the effective value for N_{loci} is only changed slightly from its original value of 2.5. Hence, a mean production rate per locus of approximately once per 20 min is retained.

Above we found a linear scaling of the total mRNA production rate with cell volume (Figure 2): $p(V) = \beta V d$. Since the total production rate is the sum of the production rates at the individual loci, we also have $p(V) = F(V)N_{loci}(V)$, where we now explicitly include volume dependence in both the number of loci $N_{loci}(V)$ and the production rates per locus $F(V)$. Rearranging, we conclude that $F(V) = \frac{\beta V d}{N_{loci}(V)}$. Unfortunately, however, the relationship $N_{loci}(V)$ between gene copy number and cell volume remains unclear. Nevertheless, by using 2 and 4 as respective lower and upper bounds on the gene copy number $N_{loci}(V)$, we can estimate bounds on the average production rates per locus for a given volume. For small cells (V = 0.5 pL), these bounds are once per 135 min and 65 min. In large cells (V = 4 pL), the average production rate is increased to lie between once per 16 min and 8 min.

In our analysis above, we have extracted mRNA production rates per locus. Such a production rate can be interpreted as a transcription initiation rate provided there is no premature Pol II termination or co-transcriptional degradation of the transcripts. Previous work has failed to find evidence for such processes at *FLC* in high transcriptional states such as those investigated here (Wu et al., 2016). We therefore interpret our mRNA production rates per locus as transcription initiation rates, with a mean value of approximately once per 20 min per locus.

Finally, we also revisited the ON/OFF model from the previous section to investigate whether bursty transcription dynamics at each locus could be consistent with our cellular mRNA distribution. Here, we used the above mRNA production rate per locus $F(V)$, using the lower (upper) bound values of N_{loci} = 2 (4) (STAR Methods). The cellular mRNA levels are the sum over N_{loci} independent simulation outcomes. We then repeated our earlier analysis to find again that a burst size per locus of at most three transcripts remained consistent with our observed mRNA distribution for both N_{loci} = 2 and 4 (STAR Methods). These results reconfirm that *FLC* transcriptional dynamics are inconsistent with large transcriptional bursts.

Simultaneous Quantification of Pol II Elongation, Intron 1 Processing, and Lariat Degradation

To further quantitate any relation between cell size and the transcriptional and RNA processing dynamics at *FLC*, we next investigated *FLC* intron 1 levels more extensively using smFISH. Generally intronic RNA levels depend on Pol II initiation (F, unit: s^{-1}), Pol II elongation (v, unit: bp/s), and intron processing (σ, unit: s^{-1}) (Figure 3A). The timescale σ^{-1} equates to the time interval between completion of intron 1 transcription and the start of lariat degradation, and thus includes the acts of splicing and lariat debranching. Intronic RNA levels are additionally dependent on lariat degradation. Since it is unclear how lariat degradation occurs (Hesselberth, 2013), we allow for both 5′ to 3′ and 3′ to 5′ degradation, with rates k_{53} and k_{35}, respectively (unit: bp/s). Potential other RNA degradation mechanisms, e.g., endoribonuclease cleavage, could be captured through large values of either or both of the degradation rates described above. However, as shown below, we find little evidence for such scenarios at *FLC*.

To quantify these processes we developed a new methodology, measuring various different intronic RNA levels and using these measurements to determine the above kinetic parameters. Specifically, we designed four different smFISH probe sets (Figure 3B) covering, respectively: the full intron 1 as described above (Figure 2A), the 5′ half, the middle of the intron (symmetrically positioned with a length of half the intron), and the 3′ half.

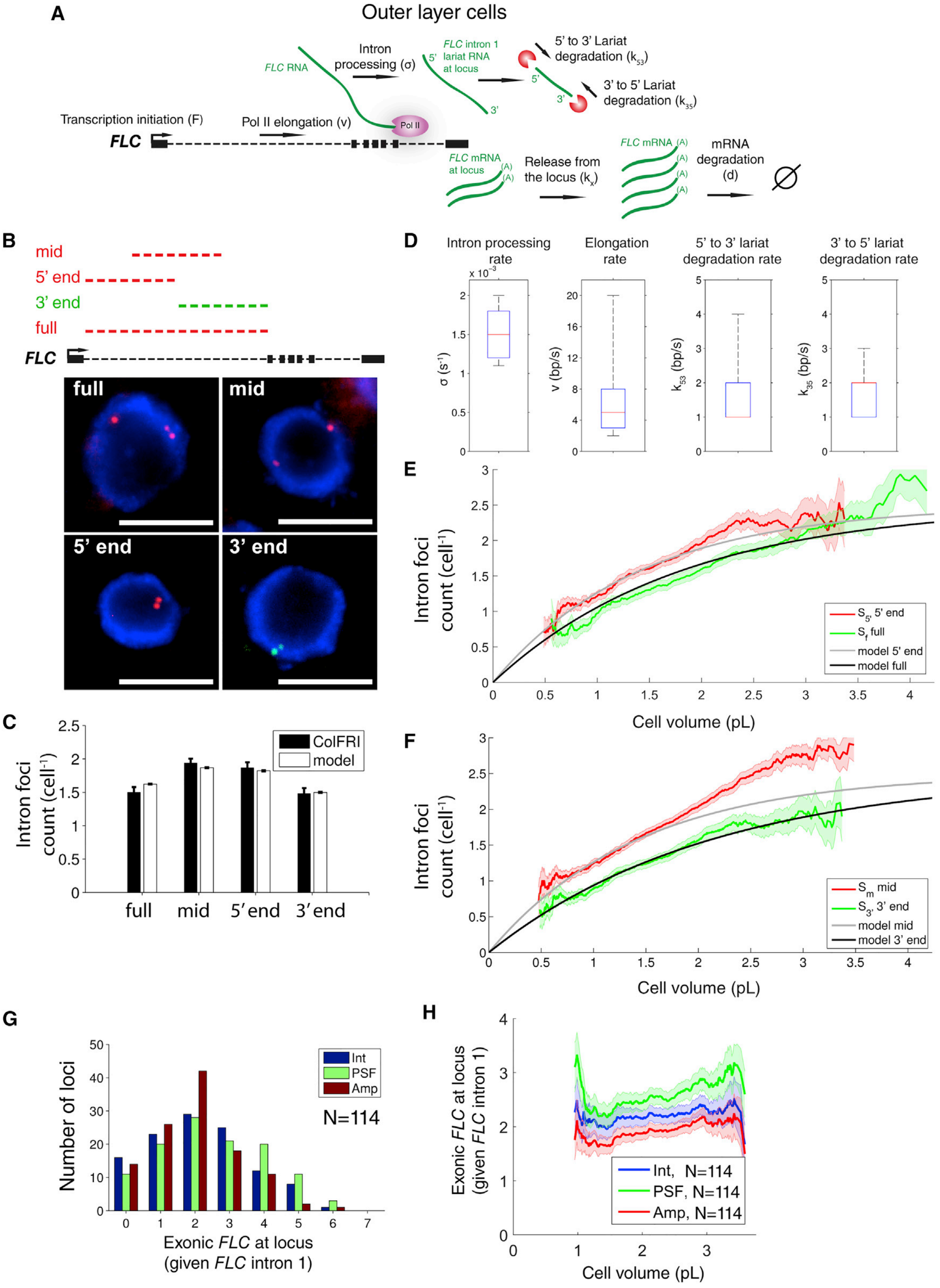

Figure 3. Quantification of Pol II Elongation, *FLC* Intron 1 Processing, Lariat Degradation, and mRNA Release from the Locus

(A) Schematic of different processes contributing to *FLC* intron 1 life cycle: transcription initiation rate (F), Pol II elongation (v), and intron processing rate (σ). Time scale σ^{-1} indicates the time interval between completion of intron 1 transcription and start of lariat degradation, which can occur from 5′ to 3′ end (rate: k_{53}) and/or 3′ to 5′ end (rate: k_{35}). Also shown is mRNA release from the locus (rate: k_x) and subsequent degradation (rate: d).

(B) Fluorescence localization (z-stack projection) of four different *FLC* intron 1 probe sets, as indicated on schematic: full-length (full, red), 5′ end (red), middle (mid, red), and 3′ end (green). All images are overlays of respective intron 1 signal and DAPI stain (blue) in representative outer layer root cells. Scale bar, 5 μm.

(legend continued on next page)

We denote their respective mean RNA levels at an *FLC* locus as I_f, $I_{5'}$, I_m and $I_{3'}$. For all of the probe sets, the mean RNA levels are then described by $I_j = FT_j$. F is the average *FLC* transcription initiation rate per locus as estimated above and T_j is the mean lifetime of the intronic RNA, which is specific for each probe set location (subscript $j = \{f,5',m,3'\}$). Importantly, for each probe set, the respective lifetime T_j depends on the kinetic parameters (STAR Methods). This timescale is additionally dependent on how many probes need to be bound for a signal to be experimentally detected. We and others have found that having only two-thirds of the probe set bound could already be sufficient to generate a detectable signal (Duncan et al., 2016; Raj et al., 2008; Rosa et al., 2016). Consequently, intronic RNA with a "missing length fraction" of up to one-third of the probe set length is assumed to still generate a detectable smFISH signal (STAR Methods).

To quantify the four unknown kinetic rates (v, σ, k_{35}, and k_{53}), we determined for each of the four probe sets the mean number of foci per cell, indicated respectively as $\langle S_f \rangle$, $\langle S_{5'} \rangle$, $\langle S_m \rangle$, and $\langle S_{3'} \rangle$ (Figures 3B and 3C). We found that $\langle S_f \rangle$ and $\langle S_{3'} \rangle$ were similar and likewise for $\langle S_{5'} \rangle$ and $\langle S_m \rangle$ (Figure 3C). Consistent with $\langle S_{5'} \rangle$ being larger than $\langle S_{3'} \rangle$ (Figure 3C), all 3′ foci co-localized with 5′ foci but not the reverse. A positive signal corresponds to at least one intron RNA molecule, which co-localizes with the *FLC* locus exclusively (Figure S1A). Therefore, the mean foci number per cell equals the average number of *FLC* loci per cell multiplied by the expectation to observe a signal at a locus (STAR Methods): $\langle S_j \rangle = N_{loci}(1 - e^{-FT_j})$.

Here, we use Poisson transcription initiation and RNA degradation kinetics, consistent with our findings above. To determine which values for the a priori unknowns (v, σ, k_{35}, and k_{53}) generated a good fit to the average foci per cell for each probe set, we systematically varied these four parameters, calculated the respective model values for the average foci per cell, and assessed with a χ^2 test (degrees of freedom k = 4, acceptance probability $p \geq 0.1$) whether the model values were sufficiently probable compared with the experimental data in Figure 3C. As a result, we obtained distributions of parameter values that generated good model fits (Figure 3D), with all the consistent parameter estimates lying in the range v = 2–20 bp/s, σ = 1.0–2.0 × 10^{-3} s^{-1}, k_{53} = 1–5 bp/s, and k_{35} = 1–4 bp/s. To determine the robustness of our parameter estimates to uncertainty in the missing length fraction, we repeated the parameter inference also for a range of missing length fractions (STAR Methods). The resulting variation in our kinetic rate estimates was limited (Figures S2A–S2D), yielding similar estimates to the above methodology. Our elongation rate (0.1–1.2 kb/min) and intron processing timescale estimates (8–17 min) are in line with the ranges described for other species (Bentley, 2014).

We next wondered whether the four above kinetic rates might scale with cell size, similar to the transcription initiation rate. To investigate this question, we first examined how the number of intron foci scaled as a function of cell size by sorting and including cells according to their size (STAR Methods). We found that levels of intronic RNA from all four probe sets $\langle S_j(V) \rangle$ increased systematically with increasing cell size (Figures 3E, 3F, and S2E), as would be expected with a transcription initiation rate that also increases with cell size.

To investigate whether we could explain these cell size dependencies with our above determined kinetic rate estimates, we first generalized the model prediction for the foci number for a given volume V: $\langle S_j(V) \rangle = N_{loci}(V)(1 - e^{-F(V)T_j(V)})$. We next assumed that only the transcription initiation rate varied with size: $F(V) = \frac{\beta V d}{N_{loci}}$, with the size-independent probe specific lifetimes $T_j(V) = T_j$ as determined above. To account for the unknown behavior of $N_{loci}(V)$, we adopted a similar approach as previously, first using the mean N_{loci} = 2.5, before investigating N_{loci} = 2 and N_{loci} = 4 as lower and upper bounds. Using N_{loci} = 2.5, we were able to reproduce the observed number of foci over the range of observed volumes (see Figures 3E and 3F with v = 3 bp/s, σ = 1.5 × 10^{-3} s^{-1}, k_{53} = 2 bp/s, and k_{35} = 2 bp/s). However, fitting using the lower and upper bounds N_{loci} = 2 and N_{loci} = 4, for both small volumes (0.9 pL) and large volumes (2.8 pL), yielded fits with 3-fold or more variation in the parameter values for v, σ, k_{53}, and k_{35} from those given above. Given this spread, we cannot rigorously conclude whether the co-transcriptional parameters v, σ, k_{53}, and k_{35} depend on cell size. Nevertheless, by analyzing intronic RNA foci we have been able to extract robust mean values for the *FLC* elongation, intron processing, and lariat degradation.

Quantification of *FLC* mRNA Release from the Locus

The release of mRNA from a transcribed locus is an important part of the RNA life cycle (Figure 3A) as it can influence transcript fate (Stuparevic et al., 2013). To quantify *FLC* mRNA release from the locus, we assessed the exonic *FLC* RNA distribution, i.e., *FLC* exonic sequences present at the *FLC* locus itself

(C) Average foci counts per cell for the four probe sets described in (B) from outer layer cells together with analytical model fits (mean and SEM using all allowed parameter values, see text and STAR Methods). Number of cells analyzed, respectively, for full, mid, 5′, and 3′: 200, 382, 326, and 326 pooled from three, four, four, and four biological replicates, respectively. Error bars denote SEM.

(D) Marginal distributions for intron processing (σ), elongation (v), 5′ to 3′ lariat degradation (k_{53}), and 3′ to 5′ lariat degradation (k_{35}) rates that generate good fits to the data shown in (C) according to a χ^2 test (degrees of freedom k = 4, acceptance probability $p \geq 0.1$) with the "missing" length fraction 1/3. Box plots indicate minimum, 25% quantile, median, 75% quantile, and maximal values.

(E) Volume dependence of average cellular foci counts per cell in outer layer cells for *FLC* intron 1: full-length (full) and 5′ end. Number of cells analyzed as in (C). Also shown are analytical model fits (black, gray) with parameter values that generated good fits to population averages in (C): v = 3 bp/s, σ = 1.5 × 10^{-3} s^{-1}, k_{53} = 2 bp/s, and k_{35} = 2 bp/s. Error lines denote SEM as function of volume.

(F) As in (E) but for middle (mid) and 3′ end *FLC* intron 1 probe sets.

(G) Locus-associated exonic *FLC* RNA (R_{loc}) distributions given presence of full-length *FLC* intron 1 signal from outer layer cells. Three different quantification methods were used on the same experimental dataset (n = 114 cells pooled from four biological replicates): integration of 2D Gaussian fit to the spatial intensity profile (Int), superposition of point spread functions (PSF), and superposition of amplitudes of PSF at the locus (Amp) (STAR Methods; Mueller et al., 2013).

(H) Volume dependence of average locus-associated exonic *FLC* RNA levels. Data and three different quantification methods as described in (G). Error lines denote SEM as a function of volume.

OPEN
ACCESS
CellPress

Table 1. Overview of Kinetic Rates and Quantities Estimated in This Study

Symbol	Definition	Estimate
R	cellular *FLC* mRNA counts (in outer layer cells)	average: 58 ± 2; min–max: 7–145
V	cell volume (in outer layer cells)	average: 1.8 pL; min–max: 0.5–4 pL
β	slope of linear relationship between cellular *FLC* mRNA count and cell volume	31 ± 1 pL^{-1}
d	mRNA degradation rate	3.3 ± 0.1 × 10^{-5} s^{-1}
p	cellular mRNA production rate	average: 1.8 × 10^{-3} s^{-1}; min–max: 0.5–4 × 10^{-3} s^{-1}
N_{loci}	cell cycle averaged number of *FLC* loci per cell	2.5; range: 2.4–2.8
F	cell cycle averaged *FLC* transcription initiation rate	7.5 ± 0.4 × 10^{-4} s^{-1}
v	Pol II elongation rate	2–20 bp/s
σ	intron processing rate	1.0–2.0 × 10^{-3} s^{-1}
k_{53}	intron lariat 5′ to 3′ degradation rate	1–5 bp/s
k_{35}	intron lariat 3′ to 5′ degradation rate	1–4 bp/s
R_{loc}	*FLC* mRNA levels at locus	average: 2.2 ± 0.3; min–max: 0–6
k_x	mRNA release rate from locus	3.4–5 × 10^{-4} s^{-1}
bs	burst size in ON/OFF model	1–3
k_{off}	transition rate from ON to OFF state in ON/OFF model	0.1 s^{-1}

(Figure 2A). By utilizing the software package FISH-quant (Mueller et al., 2013), we performed an unbiased quantitative image analysis on the *FLC* exonic intensities at *FLC* loci. First, a systematic averaging of *FLC* exonic smFISH signal (Figure 2A) spatial intensity profiles resulted in the point spread function (PSF) that represents a single *FLC* RNA molecule (Mueller et al., 2013). Next we exploited the full-length *FLC* intron 1 probe set to label the *FLC* locus itself (Figures 2A and S1A). The exonic *FLC* RNA signals co-localizing with intron 1 were then quantitatively compared with the PSF intensity using three different quantification algorithms (STAR Methods; Mueller et al., 2013) each estimating how many exonic RNAs are at the locus (Figure 3G). All three methods gave similar exonic *FLC* RNA distributions (Figure 3G), with up to at most six transcripts at the locus in all three methods, which indicates the robustness of this approach. We estimate the average exonic *FLC* RNA at the locus (conditioned on the presence of intron 1 *FLC*) to be $\langle R_{loc} \rangle = 2.2 \pm 0.3$.

To quantify the mean exonic *FLC* RNA release rate from the locus, we first assume that the mean total release is equal to $k_x \langle R_{loc} \rangle$, where k_x is the release rate. Equating this to the mean initiation rate F, we find that $k_x = 3.4 \times 10^{-4}$ s^{-1}. However, this analysis overlooks any possible correlations between the presence of exonic and intronic signal. Since our measurements of exonic RNA are conditioned on the presence of intronic RNA signal, such correlations might alter our estimate for k_x. To quantitatively investigate this possibility, we performed stochastic simulations of the transcriptional dynamics at an individual *FLC* locus using a spatiotemporal Gillespie algorithm (Figures 3A and S3A; STAR Methods). This methodology simulated the spatiotemporal transcriptional dynamics at *FLC*, including *FLC* mRNA release from the locus as a single-step Poisson process with an a priori unknown probability per unit time k_x. This analysis generated results that could then be compared with the experimental exonic *FLC* RNA distributions. We used the kinetic parameters determined previously (v = 3 bp/s, $\sigma = 1.5 \times 10^{-3}$ s^{-1}, k_{53} = 2 bp/s, k_{35} = 2 bp/s) to simulate an *FLC* locus in a cell of average size $\langle V \rangle$ = 1.8 pL, where the mean transcription initiation probability per unit time was given by $F = \frac{\beta \langle V \rangle d}{N_{loci}}$, with β = 31 pL^{-1}, $d = 3.3 \times 10^{-5}$ s^{-1},and N_{loci} = 2.5, assuming also a missing length fraction of 1/3 for all smFISH species. As expected, with this procedure we could reproduce the mean levels of the *FLC* mRNA as well as the four different intron 1 probe sets. We next output the simulated distributions of exonic RNA at the locus given that at least one intron was present at the locus and fitted k_x, the mean release probability per unit time, through the mean value of the experimental exonic RNA distribution $\langle R_{loc} \rangle$. This resulted in $k_x = 5 \times 10^{-4}$ s^{-1}, approximately once per half an hour, similar to our earlier estimate.

We next checked that our Gillespie algorithm simulations could account for our earlier data on intron foci number as a function of cell size. Using a size-dependent transcription initiation probability per unit time per locus of $F(V) = \frac{\beta V d}{N_{loci}}$, we could again reproduce these data satisfactorily (Figure S3B), confirming our earlier analytic approach.

Finally, we measured experimentally the amount of exonic RNA at the locus as a function of cell size (Figures 3H and S3C). Notably, this quantity varied only weakly with cell size, quite unlike the intronic foci signal (Figures 3E and 3F). This result suggests that the RNA release rate from the locus should scale linearly with cell size in order to compensate for a similarly increasing transcription initiation rate, thereby generating an approximately constant exonic signal. Overall, we have succeeded in quantitating the size scaling of total mRNA production and the size-independent mRNA degradation rate, as well as the mean rates for transcription elongation, intron processing, lariat degradation, and mRNA release from the locus (Table 1).

Antisense Transcription Contributes to a Decrease of Intronic *FLC* Levels with Cell Size in Prevasculature Cells

FLC expression is quantitatively repressed by the Autonomous pathway, in a mechanism involving antisense (*COOLAIR*)

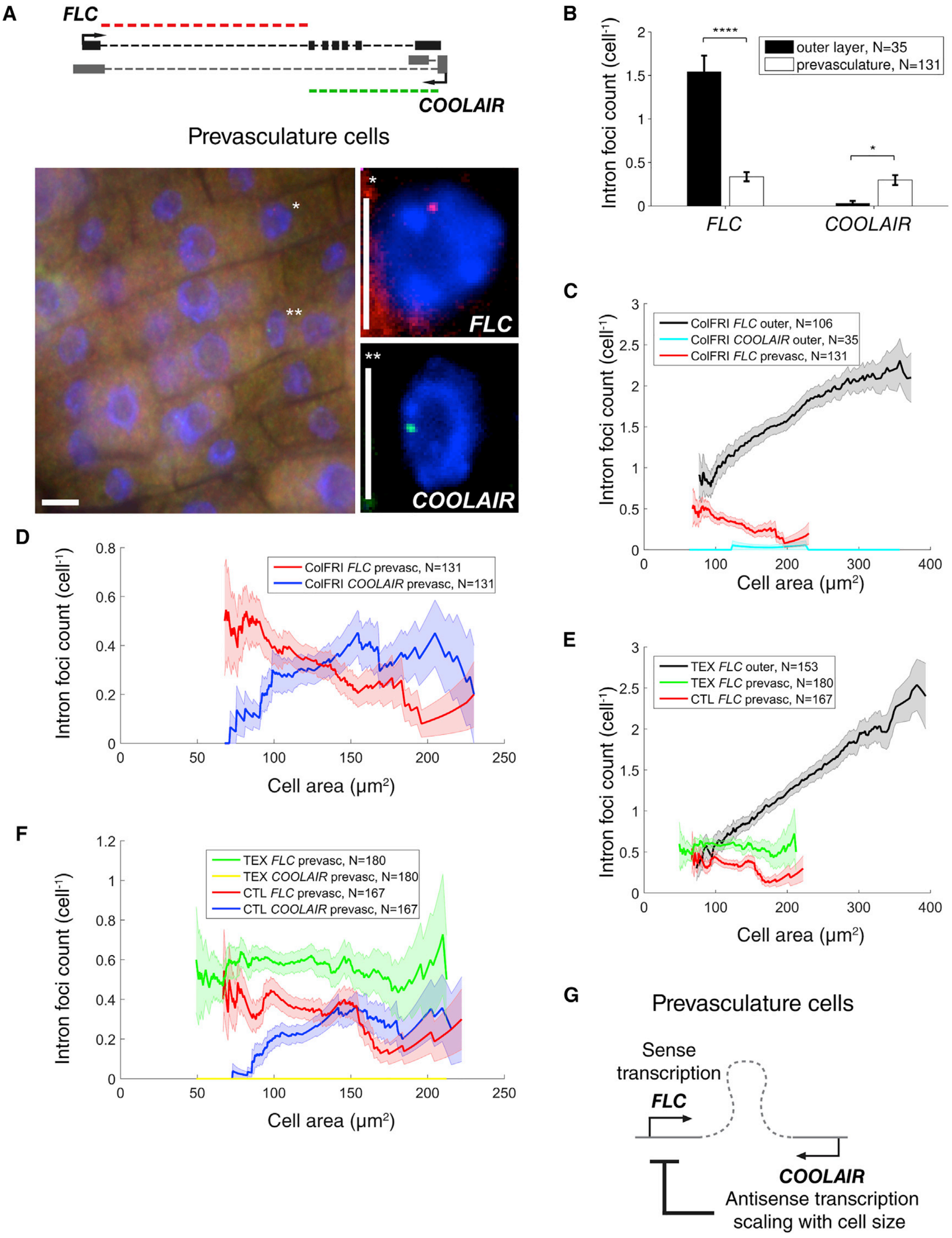

Figure 4. Antisense Transcription Contributes to a Reduction of *FLC* Intron 1 Expression with Cell Size in Prevasculature Tissue

(A) Fluorescence localization of full-length sense intron 1 (red, top right) and antisense (*COOLAIR*) 5′ end distal intron (green, bottom right) and wider-field merge (left), all with DAPI stain (blue), in representative ColFRI prevasculature root cells, with the same cells in different images identified by * or **, respectively. Scale bar, 5 μm. Also shown (top) is schematic of *FLC* gene with sense transcript as in Figure 2A, and antisense proximal and distal isoforms together with corresponding sense and antisense probe sets.

(B) Average cellular foci number of full-length *FLC* intron 1 and *COOLAIR* 5′ end distal intron in outer layer (black) and prevasculature cells (white), from one and three biological replicates, respectively. Error bars denote SEM. *$p < 0.05$, ****$p < 10^{-4}$.

(C) Area dependence of average cellular foci count per cell of full-length *FLC* intron 1 and *COOLAIR* 5′ end distal intron in ColFRI root outer layer cells (outer) and full-length *FLC* intron 1 in prevasculature (prevasc) root cells. Data as in (B) and Figure 2B. Error lines denote SEM as a function of area.

(legend continued on next page)

transcription. The *COOLAIR* transcription start site is located immediately downstream of the sense *FLC* poly(A) site (Swiezewski et al., 2009) (Figure 4A). In the root outer layer (epidermis and cortex) cells studied above (Figure 1), from where our estimates for *FLC* transcriptional kinetics are extracted, we detected no antisense *COOLAIR* expression (Figure 4B). To investigate the role of antisense transcription on sense regulation, we therefore shifted our focus onto the inner prevasculature cells (Figure 1), which show higher levels of *COOLAIR* transcription (Figure 4B) (Rosa et al., 2016).

Due to the dense packing of prevasculature cells (Figure 4A), accurate cellular volume and *FLC* mRNA estimates could not be obtained. This difficulty precluded quantification of transcriptional kinetics in these cells. However, cell area as well as sense and antisense intronic smFISH foci (Figure 4A) could still be determined manually, the small number of intron signals being much easier to definitely assign to cells, as compared with the much larger number of *FLC* mRNA foci. With this approach, it was therefore still possible to investigate the cell size dependencies of sense and antisense transcription using the intronic signals.

Average cellular *FLC* intron 1 foci counts were lower in prevasculature tissue than in the root outer layer (Figure 4B), partly because prevasculature cells are smaller (Figure 4C). As expected, in outer layer cells, *FLC* intron 1 foci counts increased with cell size, consistent with transcription initiation increasing with cell size in these cells. However, in prevasculature cells the intron 1 foci counts generally reduced with cell size (Figure 4C). Moreover, using probes at the 5′ end of the distal *COOLAIR* intron (Figure 4A), *COOLAIR* foci counts increased with cell size in the prevasculature cells (Figure 4D). We found previously that *COOLAIR* and *FLC* expression are mutually exclusive at single *FLC* loci (Figure S4 and Rosa et al., 2016). Consistently, we find here that mean sense/antisense levels as a function of cell size in prevasculature tissue are strongly anticorrelated (Figure 4D, Pearson correlation coefficient $r = -0.9$, $p = 10^{-73}$). To investigate whether *COOLAIR* transcription caused the reduction in sense intronic RNA levels with cell size, we measured sense intronic RNA foci counts in plants with reduced antisense expression using a previously described *FLC* Terminator EXchange (*FLC*-TEX) transgene (Csorba et al., 2014; Rosa et al., 2016). This transgene exchanges the *FLC* terminator/*COOLAIR* promoter with the strong *RBCS* terminator from *Arabidopsis RBCS3B*, acting to reduce antisense expression. As expected, in outer layer cells without observable antisense expression in ColFRI (Figures 4B and 4C), *FLC* intron 1 foci in *FLC*-TEX still increased with cell size (Figure 4E). Moreover, compared with an *FLC* control (*FLC*-CTL) transgene (Csorba et al., 2014; Rosa et al., 2016), antisense expression was now almost absent in prevasculature tissue in *FLC*-TEX (Figure 4F). In these cells, *FLC* intron 1 RNA foci counts were now increased (Figure 4F), most notably in larger cells where antisense expression was highest in ColFRI (Figure 4D) and *FLC*-CTL (Figure 4F). This result strongly favors a causal role for *COOLAIR* in repressing sense transcription. However, sense intron 1 RNA foci counts in *FLC*-TEX prevasculature cells did not revert to increasing with cell size, as in the outer cells, but were instead roughly constant with cell size (Figure 4F). We conclude that in prevasculature cells *COOLAIR* expression increases with cell size and that *COOLAIR* contributes to repression of sense *FLC* transcription (Figure 4G). As a result, sense *FLC* expression in prevasculature cells reduces with increasing cell size.

DISCUSSION

In this work, we have dissected with high resolution the cell-size-dependent RNA kinetics and variability of *FLC*, an important developmental regulator in a multicellular eukaryote. Our analysis has indicated that in outer layer cells without observable *COOLAIR* expression, total cellular mRNA production increases linearly with cell size, while the mRNA degradation rate is cell size independent. In these cells, we also quantified the mean rates for transcription elongation, intron processing, and lariat degradation, as well as mRNA release from the locus (Table 1) using a new methodology measuring intronic RNA levels. The net result of these dependencies is a cytoplasmic *FLC* mRNA number that scales linearly with cell size (Figure 2G). We also found low levels of stochasticity at *FLC*, with little evidence for bursty transcription.

The low levels of stochasticity at *FLC* do not support the conventional picture of bursty gene regulation. Instead, the apparent broad width of the cellular *FLC* mRNA distribution is almost entirely explained by a linear scaling of total mRNA production with cell size. This striking conclusion suggests that previous work on stochastic gene expression may need to be revisited, more carefully controlling for sources of deterministic variation between cells, as recently argued for mammalian cells (Battich et al., 2015; Kempe et al., 2015; Padovan-Merhar et al., 2015). Potentially, the large cell size diversity (~8-fold) predisposes this feature as being particularly important in plants. When controlling for cell size, the residual *FLC* mRNA variation is then consistent with Poissonian transcription initiation dynamics. Limited bursty transcription kinetics are also consistent with our data but where the burst size is at most only three transcripts. These conclusions are supported by the low levels of transcript accumulation observed at *FLC* loci and are also compatible with a burst size of about five transcripts found for a number of endogenous mammalian genes (Levesque and Raj, 2013). Importantly, transcriptional bursts, often modeled as purely stochastic events, could arise not only due to intrinsic noise in biochemical reactions, but also through deterministic sources of cell-to-cell or time-dependent variation (Sherman et al., 2015; Zopf et al., 2013). Altogether, our findings significantly advance our understanding of gene regulation in plants, where issues such as transcriptional scaling with cell size and

(D) As in (C), here for *FLC* and *COOLAIR* expression in prevasculature cells.

(E) Area dependence of average cellular foci counts per cell of full-length *FLC* intron 1 from terminator exchange (TEX) or wild-type *FLC* transgenes (CTL), in outer layer (outer) or prevasculature (prevasc) root cells, all from four biological replicates per condition. Error lines denote SEM as a function of area.

(F) As in (E), here for *FLC* and *COOLAIR* 5′ end distal intron expression in prevasculature cells.

(G) Schematic of *FLC* regulation by cell-size-dependent *COOLAIR* transcription in prevasculature cells.

stochastic or bursty gene expression have not previously been investigated.

A further key outcome of our analysis is quantification of transcription initiation and elongation rates for *FLC*. We found that in an outer layer cell of average size, the typical time between initiation events (~20 min) is similar to the time to elongate (5–50 min) through the gene. Previous work has indicated that the *FLC* locus adopts a 5′ to 3′ end looped configuration (Crevillen et al., 2013). We propose as a possible scenario that in outer layer cells, which lack observable antisense expression, a single Pol II could transcribe the sense strand almost continuously and reinitiate soon after termination. This mechanism could also explain the apparent absence of large transcriptional bursts at *FLC*. Furthermore, intron processing and degradation of *FLC* intron 1 (~3.5 kb in length) takes a considerable time (15–45 min) and appears to occur on a similar timescale as Pol II elongation through the gene. Our estimates are also consistent with recent findings in budding yeast that splicing (exon/exon ligation) occurs mostly up to 50 bp downstream of the intron acceptor site (Carrillo Oesterreich et al., 2016). With our Pol II elongation rates, this would be within a minute and thus much shorter than our intron-processing timescale (8–17 min), which reflects the timescale from intron birth until the start of lariat degradation. Overall, our transcription and RNA-processing kinetic estimates indicate that these processes occur in parallel and with relatively similar timescales.

We have found that in prevasculature tissue, cell-size-dependent *COOLAIR* transcription represses sense expression. This contributes to an overall decrease of sense *FLC* transcription with cell size in these cells in contrast to the positive scaling observed in outer layer cells. Interestingly, it is antisense transcription that positively correlates with size in prevasculature tissue, indicating that its expression could be prioritized over sense transcription in these cells. The mechanistic basis for such prioritization remains unclear, but is an intriguing question for future investigation both at *FLC* and genome-wide. Our previous whole plant studies showed that co-transcriptional proximal *COOLAIR* processing contributes to *FLC* repression (Marquardt et al., 2014; Wang et al., 2014), likely through a repressed chromatin state at the locus (Liu et al., 2010; Wu et al., 2016). Distally polyadenylated *COOLAIR* also influences transcription of the sense *FLC* strand (Li et al., 2015). How nascent *COOLAIR* transcription in prevasculature cells connects with these mechanisms remains to be addressed. Nevertheless, given the widespread prevalence of antisense expression in different organisms (Lenstra et al., 2015; Mayer et al., 2015; Pelechano and Steinmetz, 2013), antisense transcription could be a general mechanism to modulate size scaling of transcription.

Overall, our precise quantification of the *FLC* RNA kinetics is a vital step in the functional dissection of *FLC* regulation. In the future it will be interesting to see how quantitative *FLC* kinetics are modulated by temperature, ranging from the relatively high temperatures (20°C) analyzed here to winter cold (4°C). In the latter case the vernalization pathway is activated, which represses *FLC* transcription at a single-cell level via the Polycomb system in a digital and epigenetically stable manner (Angel et al., 2011; Berry and Dean, 2015; Song et al., 2012). How quantitative *FLC* regulation in the warm interfaces with digital vernalization is a critical matter for future investigation. Such investigations are expected to benefit from the single-cell kinetic quantification methods developed in this study.

STAR★METHODS

Detailed methods are provided in the online version of this paper and include the following:

- KEY RESOURCES TABLE
- CONTACT FOR REAGENT AND RESOURCE SHARING
- EXPERIMENTAL MODEL AND SUBJECT DETAILS
  - Species and Genotype of Experimental Models
  - Plant Material and Growth Conditions
- METHOD DETAILS
  - smFISH Procedure on Root Squashes
  - Synthesis of the Probes
  - Image Acquisition
  - Combined RNA-DNA FISH
- QUANTIFICATION AND STATISTICAL ANALYSIS
  - smFISH RNA Count Quantification
  - Estimation of Cellular Volumes and Areas
  - Calculation of Mean and Standard Error of Parameter Estimates Using Propagation of Errors
  - Poisson Process Describing *FLC* mRNA Degradation
  - Mathematical Characterization of Variation Due to Cell Volume and Intrinsic Noise
  - Time Averaging of *FLC* Gene Copy Number throughout the Cell Cycle
  - Determination of Cellular *FLC* Intron 1 and Exonic Foci Count Scaling with Cell Size
  - Inference of Pol II Elongation, *FLC* Intron 1 Processing and Lariat Degradation Rates
  - Stochastic Simulations of Cellular *FLC* mRNA Production and Degradation
  - Stochastic Simulations of Spatiotemporal *FLC* Transcriptional Dynamics
  - Quantification of *FLC* mRNA Release from the Locus
- DATA AND SOFTWARE AVAILABILITY

SUPPLEMENTAL INFORMATION

Supplemental Information includes four figures and one table and can be found with this article online at http://dx.doi.org/10.1016/j.cels.2017.05.010.

AUTHOR CONTRIBUTIONS

R.I., S.R., Z.W., C.D., and M.H. conceived the study; S.R. and Z.W. performed the experiments; R.I., S.R., and Z.W. performed data analysis; R.I. and M.H. constructed the mathematical models; R.I. performed the simulations; R.I., C.D., and M.H. wrote the manuscript, with assistance from S.R. and Z.W.

ACKNOWLEDGMENTS

This work was supported by BBSRC grant BB/K007203/1 (M.H., C.D.), BBSRC Institute Strategic Program GRO (BB/J004588/1), BBSRC studentship, EMBO Fellowship ALTF 422-2016 (R.I.), and European Research Council Advanced grant MEXTIM (M.H., C.D.). We thank Matthew Hartley and Tjelvar Olsson for developing the volume estimation code, Susan Duncan for help with the smFISH experiments, and Rea Kourounioti for comments on the manuscript. We thank C.D. and M.H. group members for discussions.

Received: December 19, 2016
Revised: March 5, 2017
Accepted: May 11, 2017
Published: June 14, 2017

REFERENCES

Angel, A., Song, J., Dean, C., and Howard, M. (2011). A Polycomb-based switch underlying quantitative epigenetic memory. Nature *476*, 105–108.

Battich, N., Stoeger, T., and Pelkmans, L. (2015). Control of transcript variability in single mammalian cells. Cell *163*, 1596–1610.

Bentley, D.L. (2014). Coupling mRNA processing with transcription in time and space. Nat. Rev. Genet. *15*, 163–175.

Berry, S., and Dean, C. (2015). Environmental perception and epigenetic memory: mechanistic insight through *FLC*. Plant J. *83*, 133–148.

Carrillo Oesterreich, F., Herzel, L., Straube, K., Hujer, K., Howard, J., and Neugebauer, K.M. (2016). Splicing of nascent RNA coincides with intron exit from RNA polymerase II. Cell *165*, 372–381.

Chubb, J.R., and Liverpool, T.B. (2010). Bursts and pulses: insights from single cell studies into transcriptional mechanisms. Curr. Opin. Genet. Dev. *20*, 478–484.

Corrigan, A.M., Tunnacliffe, E., Cannon, D., and Chubb, J.R. (2016). A continuum model of transcriptional bursting. Elife *5*, e13051.

Crevillén, P., and Dean, C. (2011). Regulation of the floral repressor gene *FLC*: the complexity of transcription in a chromatin context. Curr. Opin. Plant Biol. *14*, 38–44.

Crevillen, P., Sonmez, C., Wu, Z., and Dean, C. (2013). A gene loop containing the floral repressor *FLC* is disrupted in the early phase of vernalization. EMBO J. *32*, 140–148.

Csorba, T., Questa, J.I., Sun, Q.W., and Dean, C. (2014). Antisense *COOLAIR* mediates the coordinated switching of chromatin states at *FLC* during vernalization. Proc. Natl. Acad. Sci. USA *111*, 16160–16165.

Dar, R.D., Razooky, B.S., Singh, A., Trimeloni, T.V., McCollum, J.M., Cox, C.D., Simpson, M.L., and Weinberger, L.S. (2012). Transcriptional burst frequency and burst size are equally modulated across the human genome. Proc. Natl. Acad. Sci. USA *109*, 17454–17459.

de la Mata, M., Alonso, C.R., Kadener, S., Fededa, J.P., Blaustein, M., Pelisch, F., Cramer, P., Bentley, D., and Kornblihtt, A.R. (2003). A slow RNA polymerase II affects alternative splicing in vivo. Mol. Cell *12*, 525–532.

Dolken, L., Ruzsics, Z., Radle, B., Friedel, C.C., Zimmer, R., Mages, J., Hoffmann, R., Dickinson, P., Forster, T., Ghazal, P., et al. (2008). High-resolution gene expression profiling for simultaneous kinetic parameter analysis of RNA synthesis and decay. RNA *14*, 1959–1972.

Duncan, S., Olsson, T.S.G., Hartley, M., Dean, C., and Rosa, S. (2016). A method for detecting single mRNA molecules in *Arabidopsis thaliana*. Plant Methods *12*, 13.

Elowitz, M.B., Levine, A.J., Siggia, E.D., and Swain, P.S. (2002). Stochastic gene expression in a single cell. Science *297*, 1183–1186.

Gardiner, C.W. (2009). Handbook of Stochastic Methods (Springer).

Hayashi, K., Hasegawa, J., and Matsunaga, S. (2013). The boundary of the meristematic and elongation zones in roots: endoreduplication precedes rapid cell expansion. Sci. Rep. *3*, 2723.

Hazelbaker, D.Z., Marquardt, S., Wlotzka, W., and Buratowski, S. (2013). Kinetic competition between RNA Polymerase II and Sen1-dependent transcription termination. Mol. Cell *49*, 55–66.

Hesselberth, J.R. (2013). Lives that introns lead after splicing. Wiley Interdiscip. Rev. RNA *4*, 677–691.

Huh, D., and Paulsson, J. (2011). Non-genetic heterogeneity from stochastic partitioning at cell division. Nat. Genet. *43*, 95–100.

Ietswaart, R., Wu, Z., and Dean, C. (2012). Flowering time control: another window to the connection between antisense RNA and chromatin. Trends Genet. *28*, 445–453.

Kempe, H., Schwabe, A., Cremazy, F., Verschure, P.J., and Bruggeman, F.J. (2015). The volumes and transcript counts of single cells reveal concentration homeostasis and capture biological noise. Mol. Biol. Cell *26*, 797–804.

Lee, I., Michaels, S.D., Masshardt, A.S., and Amasino, R.M. (1994). The late-flowering phenotype of frigida and mutations in luminidependens is suppressed in the landsberg erecta strain of *Arabidopsis*. Plant J. *6*, 903–909.

Lenstra, T.L., Coulon, A., Chow, C.C., and Larson, D.R. (2015). Single-molecule imaging reveals a switch between spurious and functional ncRNA transcription. Mol. Cell *60*, 597–610.

Levesque, M.J., and Raj, A. (2013). Single-chromosome transcriptional profiling reveals chromosomal gene expression regulation. Nat. Methods *10*, 246–248.

Li, P.J., Tao, Z., and Dean, C. (2015). Phenotypic evolution through variation in splicing of the noncoding RNA *COOLAIR*. Genes Dev. *29*, 696–701.

Liu, F., Marquardt, S., Lister, C., Swiezewski, S., and Dean, C. (2010). Targeted 3′ processing of antisense transcripts triggers *Arabidopsis FLC* chromatin silencing. Science *327*, 94–97.

Marquardt, S., Raitskin, O., Wu, Z., Liu, F., Sun, Q., and Dean, C. (2014). Functional consequences of splicing of the antisense transcript *COOLAIR* on *FLC* transcription. Mol. Cell *54*, 156–165.

Mayer, A., di Iulio, J., Maleri, S., Eser, U., Vierstra, J., Reynolds, A., Sandstrom, R., Stamatoyannopoulos, J.A., and Churchman, L.S. (2015). Native elongating transcript sequencing reveals human transcriptional activity at nucleotide resolution. Cell *161*, 541–554.

Michaels, S.D., and Amasino, R.M. (1999). FLOWERING LOCUS C encodes a novel MADS domain protein that acts as a repressor of flowering. Plant cell *11*, 949–956.

Mueller, F., Senecal, A., Tantale, K., Marie-Nelly, H., Ly, N., Collin, O., Basyuk, E., Bertrand, E., Darzacq, X., and Zimmer, C. (2013). FISH-quant: automatic counting of transcripts in 3D FISH images. Nat. Methods *10*, 277–278.

Olsson, T.S., and Hartley, M. (2016). jicbioimage: a tool for automated and reproducible bioimage analysis. PeerJ *4*, e2674.

Padovan-Merhar, O., Nair, G.P., Biaesch, A.G., Mayer, A., Scarfone, S., Foley, S.W., Wu, A.R., Churchman, L.S., Singh, A., and Raj, A. (2015). Single mammalian cells compensate for differences in cellular volume and DNA copy number through independent global transcriptional mechanisms. Mol. Cell *58*, 339–352.

Pelechano, V., and Steinmetz, L.M. (2013). Gene regulation by antisense transcription. Nat. Rev. Genet. *14*, 880–893.

Raj, A., and van Oudenaarden, A. (2008). Nature, nurture, or chance: stochastic gene expression and its consequences. Cell *135*, 216–226.

Raj, A., Peskin, C.S., Tranchina, D., Vargas, D.Y., and Tyagi, S. (2006). Stochastic mRNA synthesis in mammalian cells. PLoS Biol. *4*, e309.

Raj, A., van den Bogaard, P., Rifkin, S.A., van Oudenaarden, A., and Tyagi, S. (2008). Imaging individual mRNA molecules using multiple singly labeled probes. Nat. Methods *5*, 877–879.

Raj, A., Rifkin, S.A., Andersen, E., and van Oudenaarden, A. (2010). Variability in gene expression underlies incomplete penetrance. Nature *463*, 913–918.

Rosa, S., Duncan, S., and Dean, C. (2016). Mutually exclusive sense-antisense transcription at *FLC* facilitates environmentally induced gene repression. Nat. Commun. *7*, 13031.

Schubert, V., Klatte, M., Pecinka, A., Meister, A., Jasencakova, Z., and Schubert, I. (2006). Sister chromatids are often incompletely aligned in meristematic and endopolyploid interphase nuclei of *Arabidopsis thaliana*. Genetics *172*, 467–475.

Shahrezaei, V., and Swain, P.S. (2008). Analytical distributions for stochastic gene expression. Proc. Natl. Acad. Sci. USA *105*, 17256–17261.

Sheldon, C.C., Burn, J.E., Perez, P.P., Metzger, J., Edwards, J.A., Peacock, W.J., and Dennis, E.S. (1999). The FLF MADS box gene: a repressor of flowering in Arabidopsis regulated by vernalization and methylation. Plant Cell *11*, 445–458.

Sherman, M.S., Lorenz, K., Lanier, M.H., and Cohen, B.A. (2015). Cell-to-cell variability in the propensity to transcribe explains correlated fluctuations in gene expression. Cell Syst. *1*, 315–325.

Sidaway-Lee, K., Costa, M.J., Rand, D.A., Finkenstadt, B., and Penfield, S. (2014). Direct measurement of transcription rates reveals multiple mechanisms for configuration of the *Arabidopsis* ambient temperature response. Genome Biol. *15*, R45.

Skinner, S.O., Xu, H., Nagarkar-Jaiswal, S., Freire, P.R., Zwaka, T.P., and Golding, I. (2016). Single-cell analysis of transcription kinetics across the cell cycle. Elife *5*, e12175.

Slepoy, A., Thompson, A.P., and Plimpton, S.J. (2008). A constant-time kinetic Monte Carlo algorithm for simulation of large biochemical reaction networks. J. Chem. Phys. *128*, 205101.

Song, J., Angel, A., Howard, M., and Dean, C. (2012). Vernalization—a cold-induced epigenetic switch. J. Cell Sci. *125*, 3723–3731.

Stuparevic, I., Mosrin-Huaman, C., Hervouet-Coste, N., Remenaric, M., and Rahmouni, A.R. (2013). Cotranscriptional recruitment of RNA exosome cofactors Rrp47p and Mpp6p and two distinct Trf-Air-Mtr4 Polyadenylation (TRAMP) complexes assists the exonuclease Rrp6p in the Targeting and degradation of an aberrant messenger ribonucleoprotein particle (mRNP) in yeast. J. Biol. Chem. *288*, 31816–31829.

Swiezewski, S., Liu, F., Magusin, A., and Dean, C. (2009). Cold-induced silencing by long antisense transcripts of an *Arabidopsis* Polycomb target. Nature *462*, 799–802.

Thattai, M. (2016). Universal Poisson statistics of mRNAs with complex decay pathways. Biophys. J. *110*, 301–305.

Wang, Z.W., Wu, Z., Raitskin, O., Sun, Q.W., and Dean, C. (2014). Antisense-mediated *FLC* transcriptional repression requires the P-TEFb transcription elongation factor. Proc. Natl. Acad. Sci. USA *111*, 7468–7473.

Wu, Z., Ietswaart, R., Liu, F.Q., Yang, H.C., Howard, M., and Dean, C. (2016). Quantitative regulation of *FLC* via coordinated transcriptional initiation and elongation. Proc. Natl. Acad. Sci. USA *113*, 218–223.

Yin, K., Ueda, M., Takagi, H., Kajihara, T., Aki, S.S., Nobusawa, T., Umeda-Hara, C., and Umeda, M. (2014). A dual-color marker system for in vivo visualization of cell cycle progression in *Arabidopsis*. Plant J. *80*, 541–552.

Zenklusen, D., Larson, D.R., and Singer, R.H. (2008). Single-RNA counting reveals alternative modes of gene expression in yeast. Nat. Struct. Mol. Biol. *15*, 1263–1271.

Zopf, C.J., Quinn, K., Zeidman, J., and Maheshri, N. (2013). Cell-cycle dependence of transcription dominates noise in gene expression. PLoS Comp. Biol. *9*, e1003161.

Zwillinger, D. (1998). Handbook of Differential Equations, Third Edition (Academic Press).

OPEN ACCESS
CellPress

STAR★METHODS

KEY RESOURCES TABLE

REAGENT or RESOURCE	SOURCE	IDENTIFIER
Antibodies		
anti-DIG-fluorescein antibody	Roche	Cat#11207741910; RRID: AB_514498
Chemicals, Peptides, and Recombinant Proteins		
Digoxigenin-dUTP	Roche	Cat#11745816910
Actinomycin D	SigmaTM	Cat# A4262-2MG
Glucose oxidase	Sigma TM	Cat# G0543
Bovine liver catalase	Sigma TM	Cat# C3155
Experimental Models: Organisms/Strains		
Arabidopsis ColFRI: Col-0 ecotype with an introgressed active *FRIGIDA* allele from the Spanish San Feliu 2 (Sf2) accession.	Caroline Dean laboratory, first described in (Lee et al., 1994).	N/A
Arabidopsis FLC-TEX: transformation of 12-kb *FLC* genomic DNA fragment composed of the promoter region, gene body, and replacement of the 3′ region by *rbcs3B* terminator into the *flc-2* FRI genotype. *flc-2* is a loss-of-function *FLC* genotype, which has a deletion/rearrangement within the endogenous *FLC* gene. A representative *FLC*-TEX line (no. 577) was selected.	Caroline Dean laboratory, first described in (Wang et al., 2014)	N/A
Arabidopsis FLC-CTL: transformation of *FLC* genomic construct (15 kb of the *FLC* locus, *FLC-15*) into *flc-2* FRI background.	Caroline Dean laboratory, first described in (Csorba et al., 2014)	N/A
Oligonucleotides		
smFISH probes, see Table S1	Biosearch Technologies	N/A
Software and Algorithms		
Cellular RNA count and Z-projected cell area script	Duncan et al., 2016; Olsson and Hartley, 2016	https://github.com/ri23/FISHmodel
Cell volume estimation script	This study	https://github.com/ri23/FISHmodel
Cellular mRNA production and degradation simulations	This study	https://github.com/ri23/FISHmodel
FLC transcription and RNA processing simulations	This study	https://github.com/ri23/FISHmodel
FISH-quant	Mueller et al., 2013	https://code.google.com/archive/p/fish-quant/

CONTACT FOR REAGENT AND RESOURCE SHARING

Further information and requests for resources and reagents should be directed to and will be fulfilled by the Lead Contact, Martin Howard (martin.howard@jic.ac.uk).

EXPERIMENTAL MODEL AND SUBJECT DETAILS

Species and Genotype of Experimental Models

Arabidopsis ColFRI, first described in (Lee et al., 1994): Col-0 ecotype with an introgressed active *FRIGIDA* allele from the Spanish San Feliu 2 (Sf2) accession. *Arabidopsis FLC*-TEX, first described in (Wang et al., 2014): transformation of 12-kb *FLC* genomic DNA fragment composed of the promoter region, gene body, and replacement of the 3′ region by *rbcs3B* terminator into the *flc-2*/FRI genotype. *flc-2*/FRI is a loss-of-function *FLC* genotype, which has a deletion/rearrangement within the endogenous *FLC* gene in a ColFRI background (Michaels and Amasino, 1999). A representative *FLC*-TEX line (no. 577) was selected (Csorba et al., 2014; Wang et al., 2014). Arabidopsis *FLC*-CTL, first described in (Csorba et al., 2014): transformation of *FLC* genomic construct (15 kb of the *FLC* locus, *FLC-15*) into *flc-2*/FRI background.

Plant Material and Growth Conditions

Seeds were surface sterilized in 5% v/v sodium hypochlorite for 5 min and rinsed three times in sterile distilled water. Seeds were stratified for 3 days at 5°C before germination in a growth cabinet (Sanyo MLR-351H) in vertically oriented Petri dishes containing MS media minus glucose (16 hours light, 100 μmol m^{-2} s^{-1}, 22°C ± 1°C) for 1 week.

For Actinomycin D (ActD) experiments, plants were initially germinated in non-supplemented media for 6 days and then transferred to new plates containing ActD. Before pouring into plates, molten media was supplemented with a stock solution of ActD (1mg/ml dissolved in DMSO) to a final concentration of 20ug/ml. ActD was obtained from SigmaTM (catalogue # A4262-2MG).

METHOD DETAILS

smFISH Procedure on Root Squashes

Seedlings were removed from the media and root tips were cut using a razor blade and placed into glass wells containing 4% paraformaldehyde and fixed for 30 min. Roots were then removed from the fixative and washed twice with nuclease free 1X PBS (Thermo Scientific, Lutterworth, UK). Several roots were then arranged on a Poly-L-Lysine slide (Thermo Scientific, Lutterworth, UK) and covered by a glass coverslip (Slaughter, Uppminster, UK). The meristems were then squashed by tapping the coverslips, before each slide was submerged (together with the coverslips) for a few seconds in liquid nitrogen until frozen. The coverslips were then flipped off the slides using a razor blade and the roots were left to dry at room temperature for 30 min.

Tissue permeabilization was achieved by immersing the samples in 70% ethanol for a minimum of one hour. The ethanol was then left to evaporate at room temperature for 5 min before two washes were carried out with wash buffer (containing 10% formamide and 2x SSC). 100μl of hybridization solution (containing 10% dextran sulfate, 2x SSC and 10% formamide), with each probe set at a final concentration of 250nM, was then added to each slide. Coverslips (Slaughter, Uppminster, UK) were carefully laid over the samples to prevent evaporation of the buffer and the probes were left to hybridize at 37°C overnight in the dark.

The hybridization solution containing unbound probes was pipetted off the following morning. Each sample was then washed twice with wash buffer with the second wash left to incubate for 30 min at 37°C. 100μl of the nuclear stain DAPI (100ng/mL) was then added to each slide and left to incubate at 37°C for 30 minutes. The DAPI was removed and 100μl 2xSSC was added and then removed. 100μl GLOX buffer minus enzymes (0.4% glucose in 10mM Tris, 2X SSC) was added to the samples and left to equilibrate for 2 min. This was removed and replaced with 100μl of GLOX buffer containing the enzymes glucose oxidase and catalase, where 1μl of each enzyme (Glucose oxidase (#G0543 from Sigma) and catalase (#C3155 from Sigma)) was added to a total of 100μl of GLOX minus enzymes. The samples were then covered by 22mm x 22mm No.1 coverslips (Slaughter, Uppminster, UK), sealed with nail varnish and immediately imaged.

Synthesis of the Probes

The probes for *FLC* mRNA, full-length *FLC* intron 1 and *COOLAIR* were as described in (Duncan et al., 2016; Rosa et al., 2016). We used the online program Stellaris Probe Designer version 2.0 from Biosearch Technologies to design probe sequences for the 5′ half, 3′ half and middle of *FLC* intron 1 (Table S1).

Image Acquisition

For imaging we used a Zeiss Elyra PS1 inverted microscope, with a x100 oil-immersion objective (1.46 NA) and cooled EM-CCD Andor iXon 897 camera (512x512 QE>90%); or a Zeiss CellObserver HS system equipped with a PlanApo 1.4/100x objective, an Axiocam MRm Rev. The following wavelengths were used for fluorescence detection: for probes labeled with Quasar570 an excitation line of 561 nm was used and signal was detected at 570-640 nm; for probes labeled with Quasar670 an excitation line of 642 nm was used and signal was detected at 655-710 nm; for DAPI an excitation line of 405 nm was used and signal was detected at 420-480 nm.

Maximum projections and analysis of 3D pictures were performed using Fiji (an implementation of ImageJ, a public domain program by W. Rasband available from http://rsb.info.nih.gov/ij/).

Combined RNA-DNA FISH

For sequential RNA-DNA FISH, after imaging RNA by smFISH (protocol outlined above), coverslips were removed and washed three times in 4X-SSC/0.2% Tween at 37°C. Slides were then re-fixed with 4% (w/v) paraformaldehyde (PFA) in 1XPBS buffer for 10 min and washed again several times in 1X PBS. Afther that slides were treated with 100 μg/mL RNase for 1 h at 37°C and washed twice in 1X PBS. Samples were then digested in a mixture of 1% driselase, 0.5% cellulase, and 0.025% pectolyase for 10 min at 37°C. Slides were then washed and re-fixed with 4% PFA for 10 min and transferred to a series of ethanol steps increasing to 70%, 85% and 100%.

Probes were labeled with Digoxigenin-dUTP (#11745816910, Roche) by nick translation. Bacterial artificial chromosome (BAC) clone JAtY71K18, which contains an insert of 75 kb, was used as a probe. The hybridization mixture (20 ng/mL labeled DNA, 50% formamide, 10% dextran sulfate, 2X SSC, 1mg/ml salmon sperm (D9156, Sigma)) was denatured at 85°C for 10 min and applied to the slides. Slides containing the hybridization mixture were denatured for 7 min at 75°C (in an omnislide), and hybridized overnight

at 37°C. After hybridization, slides were washed at 42°C once in 2X SSC, twice in 20% formamide plus 0.1X SSC and twice in 2X SSC, and finally twice in 2X SSC at room temperature and twice in 4X SSC plus 0.2% Tween-20. Then the slides were blocked in TNB (0.1M TrisHCl, 0.15M NaCl, 3% BSA) for 30 min at 37°C. Digoxigenin–dUTP probes were detected with anti-DIG-fluorescein antibody (#11207741910, Roche) prepared in TNB buffer (1:100). Nuclei were counterstained with 1 mg/mL DAPI, and slides were mounted in Vectashield (Vector Laboratories). In order to find the cells previously imaged for smFISH, we saved the stage positions of the cells imaged and acquired large image tiles in order facilitate the identification of the cells.

QUANTIFICATION AND STATISTICAL ANALYSIS

smFISH RNA Count Quantification

Cellular count quantification of *FLC* mRNA dots from the z projection of optical sections of outer layer cells consisted of two components - segmentation and mRNA counting, as described in (Duncan et al., 2016). These two components were combined into an overall workflow that resulted in an image where each cell was annotated with the number of probes located inside it. The image analysis workflow operated on image collections where each image represented a unique channel/z-stack pair. To separate the captured microscopy image into individual channel/z-stack pairs, Bioformats was used and the pipeline was implemented in the Python programming language, available at https://github.com/JIC-CSB/FISHcount and https://github.com/ri23/FISHmodel. To determine the counting error we also determined mRNA counts on the same data sets using FISHquant, as described in brief in the last section below and also in (Mueller et al., 2013). Cellular levels of *FLC* intron 1 and *COOLAIR* (all probe sets) were determined manually using ImageJ or ZEN (proprietary software from Zeiss).

Estimation of Cellular Volumes and Areas

Volume estimation for outer layer cells was performed using two methods: the projection method and 3D segmentation method (Duncan et al., 2016; Olsson and Hartley, 2016). In the first method, we determined the cell area in pixels from the z-stack projection image that was also utilized to determine the cellular *FLC* mRNA counts (Duncan et al., 2016). This value was then multiplied by the average number of images along the z direction that contained cells in focus. Lastly we multiplied by the voxel size $0.1\times0.1\times0.2$ μm^3 for images generated using the Zeiss Elyra, and $0.075\times0.075\times0.1$ μm^3 for images generated by the Zeiss CellObserver. The second method determined the cell area in pixels for each z-plane using the same algorithm as described in (Duncan et al., 2016; Olsson and Hartley, 2016) that was previously used for the z-projection area calculation. Cell volume was then estimated as the sum of cell area pixels over all z-planes multiplied by the above voxel size. The two segmentation methods are implemented in the Python programming language and are available at https://github.com/ri23/FISHmodel.

For prevasculature cells, manual inspection indicated that the above described computational algorithms (Duncan et al., 2016; Olsson and Hartley, 2016) did not generate accurate segmentation results. This was because the prevasculature cells were more tightly packed in the root squash such that the cell outlines were less pronounced. Instead, by using ImageJ (ROI manager plugin), we manually segmented these cells to determine their cell area in pixels in one z-plane that was in focus with the DAPI signal. This value was then multiplied by the pixel area (0.1×0.1 μm^2 for Zeiss), resulting in the cell area. We confirmed that for outer layer cells, manual and computational cell area segmentation methods generated similar results.

Calculation of Mean and Standard Error of Parameter Estimates Using Propagation of Errors

Throughout this study we utilized the theory of propagation of errors to estimate the mean and error (SEM) on a quantity (e.g. parameter values) that is generally a function $f(X_1,.., X_n)$ of n experimentally determined quantities $X_1 \ldots X_n$ with given means $\langle X_1\rangle \ldots \langle X_n\rangle$ and errors (SEM) $dX_1 \ldots dX_n$. Assuming statistical independence and relatively small errors:

Mean of quantity of interest: $\langle f(X_1,.., X_n)\rangle = f(\langle X_1\rangle,.., \langle X_n\rangle)$,

Error on quantity of interest: $df(X_1,..,X_n) = \sqrt{\sum_{i=1}^{n}\left(\frac{\partial f}{\partial X_i} dX_i\right)^2}$.

Poisson Process Describing *FLC* mRNA Degradation

To model cellular *FLC* mRNA levels after transcription inhibition, we consider a Poisson process with a constant probability per unit time d for degradation of a single mRNA. The probability that a given mRNA does not degrade during a time t is therefore e^{-dt}, while the probability that it does is $1-e^{-dt}$. If the system is initialized with b mRNA molecules, then the probability $P(r,t)$ that $r \le b$ survive at time t is therefore $\binom{b}{r}(e^{-dt})^r(1-e^{-dt})^{b-r}$. Generalising to the case where the system is initialized with any number of mRNA b up to a maximum of M, with probability $P(b,0)$, then the probability $P(r,t)$ is then given by $P(r,t) = \sum_{b=r}^{M}\binom{b}{r}(e^{-dt})^r(1-e^{-dt})^{b-r}P(b,0)$, as stated in the main text.

More formally this result can also be derived as follows. The master equation for P(*r*,*t*) is given by (Gardiner, 2009): $\frac{\partial}{\partial t}\mathrm{P}(r,t) = d(r+1)\mathrm{P}(r+1,t) - d\, r\mathrm{P}(r,t)$. First, we define the generating function $G(w,t) = \sum_{b=0}^{\infty} w^b \mathrm{P}(b,t)$ (Gardiner, 2009; Shahrezaei and Swain, 2008). We then convert the master equation into a partial differential equation for $G(w,t)$: $\frac{\partial}{\partial t}G(w,t) = d(1-w)\frac{\partial}{\partial w}G(w,t)$. We solve this equation analytically for $G(w,t)$, given the Dirichlet boundary conditions $G(w,0) = \sum_{b=0}^{\infty} w^b \mathrm{P}(b,0)$, using the method of characteristics (Shahrezaei and Swain, 2008; Zwillinger, 1998). This results in the following expression: $G(w,t) = \sum_{b=0}^{\infty}(we^{-dt} + 1 - e^{-dt})^b \mathrm{P}(b,0)$. By the definition of the generating function we can make use of its Taylor series expansion to obtain $\mathrm{P}(r,t) = \frac{1}{r!}\frac{\partial}{\partial w^r}G(w,t)\Big|_{w=0}$. We then obtain the desired result: $\mathrm{P}(r,t) = \sum_{b=r}^{M}\binom{b}{r}(e^{-dt})(1-e^{-dt})^{b-r}\mathrm{P}(b,0)$.

Mathematical Characterization of Variation Due to Cell Volume and Intrinsic Noise

To assess the relation between *FLC* mRNA and volume for the data shown in Figure 2H, we investigated the mean and variances as follows. We took a bin size of $\Delta V = 0.5\ pL$ and binned the data ranging from $V_{\min} = 0.5\ pL$ up to $V_{\max} = 3.5\ pL$ accordingly. We then calculated for the data in each bin the mean (with errorbars: SEM) and variance (with error bars: standard error on the variance $SE_{Var(X)} = \frac{Var(X)}{\sqrt{n-1}}$, with *n* the number of relevant data points). Furthermore we compared these results with a model where the *FLC* mRNA scales linearly with cell volume, as described previously (Padovan-Merhar et al., 2015). We also extended this analysis to include the effects of our volume estimation error, as described below.

As described in the main text and supported by our experimental observations (Figure 2G), we consider the cellular mRNA levels as a random variable $R(V)$ such that its expectation conditioned on cell volume V is given by the observed linear relationship: $\mathrm{E}(R|V) = \beta V$ (Padovan-Merhar et al., 2015). If the residual variation would be minimal, the conditional distribution would be Poissonian: $P(R|V) = Pois(\lambda = \beta V)$. In this case, the variance as a function of volume is $Var(R|V) = \beta V$ (Figure 2H, Poisson limit). To assess the mean and variance of this Poisson model in the presence of our experimental volume measurement error of $\varepsilon = 0.3pL$ ($\approx$20% of the average cell volume, Figure S1D), we instead computed $\mathrm{E}(R(\tilde{V})\big|\tilde{V}\in[V-\varepsilon, V+\varepsilon])$ and $Var(R(\tilde{V})\big|\tilde{V}\in[V-\varepsilon, V+\varepsilon])$ as follows.

First note that by definition we have

$$\mathrm{E}\left(R\left(\tilde{V}\right)\Big|\tilde{V}\in[V-\varepsilon, V+\varepsilon]\right) = \int_0^{\infty} r\mathrm{P}\left(R\left(\tilde{V}\right) = r|\tilde{V}\in[V-\varepsilon, V+\varepsilon]\right)dr\ .$$

The conditional probability can be computed by invoking Bayes' rule:

$$\mathrm{P}\left(R\left(\tilde{V}\right) = r|\tilde{V}\in[V-\varepsilon, V+\varepsilon]\right) = \frac{\int_{V-\varepsilon}^{V+\varepsilon}\mathrm{P}(R(V) = r|V)\mathrm{P}(V)dV}{\int_{V-\varepsilon}^{V+\varepsilon}\mathrm{P}(V)dV}\ .$$

We computed in a custom-written MATLAB script, the (non-normalized) probability density P(*V*) directly from our experimental data by binning our volume estimates with bin size $\Delta V=0.02pL$. Then by inserting the underlying Poisson distribution $\mathrm{P}(R(V) = r|V) = \frac{(\beta V)^r}{r!}e^{-\beta V}$ with our experimentally observed volume distribution we can approximate the conditional probability by a sum:

$$\mathrm{P}\left(R\left(\tilde{V}\right) = r|\tilde{V}\in[V-\varepsilon, V+\varepsilon]\right) = \frac{\sum_{\tilde{V}=V-\varepsilon}^{V+\varepsilon}\frac{(\beta\tilde{V})^r}{r!}e^{-\beta\tilde{V}}\mathrm{P}\left(\tilde{V}\right)}{\sum_{\tilde{V}=V-\varepsilon}^{V+\varepsilon}\mathrm{P}\left(\tilde{V}\right)}\ .$$

With this expression we then approximated the conditional expectation and variance as sums:

$$\mathrm{E}\left(R\left(\tilde{V}\right)\Big|\tilde{V}\in[V-\varepsilon, V+\varepsilon]\right) = \sum_{r=0}^{R_{\max}} r\mathrm{P}\left(R\left(\tilde{V}\right) = r|\tilde{V}\in[V-\varepsilon, V+\varepsilon]\right)$$

and

$$\begin{aligned} &Var\left(R\left(\tilde{V}\right)\Big|\tilde{V}\in[V-\varepsilon, V+\varepsilon]\right) = \\ &\mathrm{E}\left[\left(R\left(\tilde{V}\right) - \mathrm{E}\left(R\left(\tilde{V}\right)\Big|\tilde{V}\in[V-\varepsilon, V+\varepsilon]\right)\right)^2\Big|\tilde{V}\in[V-\varepsilon, V+\varepsilon]\right] \\ &= \sum_{r=0}^{R_{\max}}\left[r - \mathrm{E}\left(R\left(\tilde{V}\right)\Big|\tilde{V}\in[V-\varepsilon, V+\varepsilon]\right)\right]^2\mathrm{P}\left(R\left(\tilde{V}\right) = r|\tilde{V}\in[V-\varepsilon, V+\varepsilon]\right) \end{aligned}$$

Here, R_{max} indicates the maximal *FLC* mRNA number as observed in our data set.

Lastly, to assess intrinsic variability in mRNA levels in the presence of external variability arising from cell volume, we computed the volume-corrected noise measure, as previously described in Ref. (Padovan-Merhar et al., 2015): $N_R := \frac{Var(R) - Var(\mathrm{E}(R|V))}{\mathrm{E}(R)^2}$. In our case of the linear relation $\mathrm{E}(R|V) = \beta V$ the second variance term can be expressed as $Var(\mathrm{E}(R|V)) = Var(\beta V) = \beta^2 Var(V)$. Furthermore by taking the expectation over $\mathrm{E}(R|V)$, we find that $\mathrm{E}(R) = \beta \mathrm{E}(V)$. We use this result to find an expression for the covariance:$Cov(R,V) = \mathrm{E}(RV) - \mathrm{E}(R)\mathrm{E}(V) = \beta \mathrm{E}(V^2) - \beta \mathrm{E}(V)^2 = \beta Var(V)$. We can now combine these results to obtain $Var(\mathrm{E}(R|V)) = \beta Cov(R,V)$. Altogether, this leads to (Padovan-Merhar et al., 2015): $N_R = \frac{Var(R)}{\mathrm{E}(R)^2} - \frac{Cov(R,V)}{\mathrm{E}(R)\mathrm{E}(V)}$. Error bars were determined by bootstrapping as in (Padovan-Merhar et al., 2015). For the Poisson limit model, we can instead calculate the volume-corrected noise measure directly from its definition. First we note that from the law of total probability (Shahrezaei and Swain, 2008; Sherman et al., 2015), we have $Var(R) = \mathrm{E}(Var(R|V)) + Var(\mathrm{E}(R|V))$. This expression then leads to the final result: $N_R = \frac{\mathrm{E}(Var(R|V))}{\mathrm{E}(R)^2} = \frac{\mathrm{E}(\beta V)}{\mathrm{E}(R)^2} = \frac{1}{\mathrm{E}(R)}$.

Time Averaging of *FLC* Gene Copy Number throughout the Cell Cycle

We used *Arabidopsis* cell cycle stage time period estimates determined by (Hayashi et al., 2013; Yin et al., 2014) to approximate the average *FLC* gene copy number N_{loci}. The cell cycle for meristematic root cells (as analysed in our smFISH assay) is, on average, 17h (Hayashi et al., 2013; Yin et al., 2014). In these cells *FLC* gene copy number increases from 2 in G1 to 4 by the end of S phase. During mitosis, transcription seems not to occur so that the copy number is effectively zero (Duncan et al., 2016; Rosa et al., 2016). If $N_{loci}(t)$ represents the gene copy number throughout the cell cycle for $0 \le t \le 17h$, then the copy number changes over time as follows:

$$N_{loci}(t) = \begin{cases} 2 & \text{if } 0 \le t \le 7h \\ 3 & \text{if } 7h \le t \le 8h \\ 4 & \text{if } 8h \le t \le 14.5h \\ 0 & \text{if } 14.5h \le t \le 17h \end{cases}$$

The time average of $N_{loci}(t)$ throughout the cell cycles is then $N_{loci} = 2.5$. As described, the cell cycle averaged production rate per locus F then equals: $F = \frac{\langle R \rangle d}{N_{loci}}$, with $\langle R \rangle$ the average cellular *FLC* mRNA level and d the mRNA degradation rate. We also varied the above dynamics such that the time points where $N_{loci}(t)$ changed (i.e. at 7h, 8h and 14.5h), could deviate by at most 1 hour from these values. Such alterations resulted in a maximal average loci value of $N_{loci} = 2.8$ and a minimum of $N_{loci} = 2.4$.

In the scenario where the production rate per locus $F(t)$ dynamically changes to counteract changes in gene copy number $N_{loci}(t)$ throughout the cell cycle time t, we would have the following situation: $C = N_{loci}(t)F(t)$, with C a constant that is not dependent on the cell cycle time (except during mitosis where $F(t)$=0). As explained in the main text, integrating over the cell cycle is equivalent to averaging over the observed cell population: $\langle R \rangle = \frac{1}{d}\frac{1}{17h}\int_0^{17h} N_{loci}(t)F(t)dt$. This results in $C = \frac{17}{14.5}\langle R \rangle d$. The time averaged production rate per locus (F) is then obtained again by integrating over the cell cycle: $F = \frac{1}{17h}\int_0^{17h} F(t)dt = \frac{\langle R \rangle d}{2.7}$. This shows that this scenario is effectively equivalent to having the cell-cycle averaged copy number $N_{loci} = 2.7$.

Determination of Cellular *FLC* Intron 1 and Exonic Foci Count Scaling with Cell Size

In order to determine how the cellular intron foci counts for the various probe sets scale with cell size (either volume or cell area), as shown in Figures 3E, 3F, and 3H, 4C–4F, S2E, S3B, and S3C, we wrote a custom MATLAB script that first ordered the cells according to their cell size. For volumes, this procedure generated the sequence $V_1 \le V_2 \ldots . \le V_N$, where N is the total number of cells. We then calculated the range of attained sizes $\langle V_k \rangle = \frac{1}{k}\sum_{i=1}^{k} V_i$ for $k = 1\ldots N$ and $\langle V_{N+k} \rangle = \frac{1}{N-k}\sum_{i=1+k}^{N} V_i$ for k=*1… N-1*, which are thus averages over the relevant cell subpopulations. Note that by construction, the $\langle V_j \rangle$ are monotonically increasing with $j = 1 \ldots (2N-1)$ and range from the minimal size $\langle V_1 \rangle = V_1$ to the maximal size $\langle V_{2N-1} \rangle = V_N$. Then, with the given cell order above, we calculate the corresponding (average) cellular intron foci counts for a given volume $\langle V_j \rangle$: $S(\langle V_k \rangle) = \frac{1}{k}\sum_{i=1}^{k} S(V_i)$ for k = *1… N*, and $S(\langle V_{N+k} \rangle) = \frac{1}{N-k}\sum_{i=1+k}^{N} S(V_i)$ for k = *1… N-1*. Error bars of these estimates are the standard error on the mean: $SE_{S(\langle V_k \rangle)} = \frac{1}{\sqrt{k}}\sqrt{\frac{1}{k-1}\sum_{i=1}^{k}(S(V_i) - S(\langle V_k \rangle))^2}$ for k = *1… N*, and $SE_{S(\langle V_{N+k} \rangle)} = \frac{1}{\sqrt{N-k}}\sqrt{\frac{1}{N-k-1}\sum_{i=1+k}^{N}(S(V_i) - S(\langle V_{N+k} \rangle))^2}$ for k = *1… N-1*. To ensure precise estimates, we have only included averages calculated from at least 10 experimentally observed cells in all plots shown.

Inference of Pol II Elongation, *FLC* Intron 1 Processing and Lariat Degradation Rates

For the estimation of the Pol II elongation rate (v), intron processing (σ) and lariat degradation rates, respectively 5′ to 3′ (k_{53}) and 3′ to 5′ (k_{53}), we investigated the levels observed from four different intron 1 smFISH probe sets (Figure 3). One covered the full intron 1, the second covered the 5′ half, the third covered the middle of the intron symmetrically, also with a length of half the intron, and the fourth covered the 3′ half. We denote their respective RNA levels at an *FLC* locus as I_f, $I_{5'}$, I_m and $I_{3'}$ (Figure 3B). The gene length covered by the 5′, mid and 3′ probe sets is L_p=1.8 kb (Table S1), i.e. half the intron length, with a covered gene length of $2L_p$ for the full-length intron probe set (Duncan et al., 2016; Rosa et al., 2016). We, and others have found that having only two thirds of the probe set bound could already be sufficient to generate a detectable focus signal (Duncan et al., 2016; Raj et al., 2008; Rosa et al., 2016). Consequently, intronic RNA with a "missing length fraction" f_{Lp} of up to one-third of the probe set length could in principle still be detected.

Full-length intron 1 has, by the definition of σ (Figure 3A), a lifetime of $T_f = \frac{1}{\sigma}$. However, detected intron 1 RNA with a missing length $2L_pf_{Lp}$ could arise from two sources: partially complete transcripts where Pol II has not yet transcribed this missing length or a previously full-length lariat RNA that is already partly degraded. Therefore, we find the expectation value: $\mathrm{E}(I_f) = \frac{F}{\sigma} + \frac{2FL_pf_{Lp}}{v} + \frac{2FL_pf_{Lp}}{k_{53}+k_{35}}$, where F is the population averaged *FLC* transcription initiation rate per locus. Effectively, the lifetime in the presence of a missing length is then $T_f = \frac{1}{\sigma} + \frac{2L_pf_{Lp}}{v} + \frac{2L_pf_{Lp}}{k_{53}+k_{35}}$.

$I_{5'}$ is constituted by full-length intron 1, but also Pol II elongating in the 3′ half of the intron. Then by taking into account again the missing length fraction arising from the same two sources: not yet fully transcribed RNA and lariat degradation intermediates, we obtain: $T_{5'} = \frac{1}{\sigma} + \frac{L_p}{v} + \frac{L_pf_{Lp}}{v} + \min\left(\frac{L_pf_{Lp}}{k_{53}}, \frac{L_p(1+f_{Lp})}{k_{53}+k_{35}}\right)$.

$I_{3'}$ is constituted by full-length intron 1, but with similar missing length fraction contributions as for $I_{5'}$: $T_{3'} = \frac{1}{\sigma} + \frac{L_pf_{Lp}}{v} + \min\left(\frac{L_pf_{Lp}}{k_{35}}, \frac{L_p(1+f_{Lp})}{k_{53}+k_{35}}\right)$.

Lastly, I_{m} is constituted of full-length intron 1, Pol II elongating in the 3′ quarter of intron 1 and the missing length fractions:

$$T_m = \frac{1}{\sigma} + \frac{0.5L_p}{v} + \frac{L_pf_{Lp}}{v} + \min\left(\frac{L_p(0.5+f_{Lp})}{k_{53}}, \frac{L_p(0.5+f_{Lp})}{k_{35}}, \frac{L_p(1+f_{Lp})}{k_{53}+k_{35}}\right)$$

.

Our observed quantities are the respective average *FLC* intron 1 foci numbers per cell: $\langle S_f \rangle$, $\langle S_{5'} \rangle$, $\langle S_m \rangle$ and $\langle S_{3'} \rangle$. We next assume that the variables I_j (subscript $j \in \{f, 5', m, 3'\}$) are Poisson distributed with mean production rate F and degradation rate T_j^{-1}. Given our average *FLC* copy number N_{loci}, the $\langle S_j \rangle$ can then be modelled by: $\mathrm{E}(S_j) = N_{loci}P(I_j{>}0) = N_{loci}(1 - \mathrm{P}(I_j = 0)) = N_{loci}(1 - \exp(-FT_j))$.

To estimate the four unknown kinetic parameters (v, σ, k_{35} and k_{53}) from our average cellular foci numbers $\langle S_j \rangle$, we wrote a custom MATLAB script that performed a systematic parameter sweep for all parameter combinations lying in the range: v=0.1-20 bp/s, k_{53}=0.1-20 bp/s and k_{35}=0.1-20 bp/s, and σ=3x10^{-4} – 20x10^{-4} s^{-1}, while we varied the missing length fraction $f_{Lp} \in \left\{0, \frac{1}{9}, \frac{2}{9}, \frac{1}{3}\right\}$.

We then calculated our chi-square statistic: $\chi^2 = \sum_{j \in \{f,5',m,3'\}} \left(\frac{\langle S_j \rangle - \mathrm{E}(S_j)}{SE_{\langle S_j \rangle}}\right)^2$. Assuming normally distributed standard errors on our estimates $SE_{\langle S_j \rangle}$, χ^2 then follows a chi-square distribution with four degrees of freedom. For all resulting χ^2 statistics, we determined their probabilities $\mathrm{P}(\chi^2)$ and accepted the statistic as a good fit when $\mathrm{P}(\chi^2) \geq 0.1$. This resulted in a set of consistent parameter sets for which the marginal distribution are shown in Figures S2A–S2D and, for $f_{Lp} = \frac{1}{3}$, in Figure 3D (boxplot: median, Q1, Q3 and min/max). Similar results were also obtained using $\mathrm{P}(\chi^2) \geq 0.05$. Note that for f_{Lp} = 0, the mathematical expressions for I_f and I_3 are equal. As a consequence, k_{53} and k_{35} remain undetermined with this missing length fraction (Figures S2C and S2D). We also performed the same procedure for a wide range of parameter values outside the above described regions, but we could not find any further good fits according to the criteria above. Lastly, we repeated the above described parameter fitting procedure to the average intron foci numbers $S_j(\langle V_k \rangle)$ (Figures 3E and 3F) for small ($\langle V_k \rangle$ = 0.9pL) and large cells ($\langle V_k \rangle$ = 2.8pL), using the lower and upper bounds N_{loci} = 2 and N_{loci} = 4 in the expression $F(\langle V_k \rangle) = \frac{\beta \langle V_k \rangle d}{N_{loci}}$ for the per locus transcription initiation probability per unit time.

Stochastic Simulations of Cellular *FLC* mRNA Production and Degradation

To investigate the potential consistency of transcriptional bursting with the observed cellular *FLC* mRNA levels (Figure 2C), we simulated stochastic cellular *FLC* mRNA production and degradation in root outer layer cells (see Figure S1E for a graphical representation) by implementing a Gillespie algorithm (Slepoy et al., 2008) in C++ (https://github.com/ri23/FISHmodel).

Cellular *FLC* mRNA production occurred with probability per unit time (propensity): $p_t = \beta V d$. This reaction resulted in a sense *FLC* mRNA: *sFLC*. This mature transcript could then be degraded with probability per unit time d. Here, $\beta = 31 pL^{-1}$ indicates the slope of the linear *FLC* mRNA scaling with cell volume V (Figure 2G). Furthermore, $d = 3.3 \times 10^{-5} s^{-1}$ indicates the experimentally determined *FLC* mRNA degradation rate.

For the ON/OFF transcription model (see Figure S1E for a graphical representation) we include reactions for the Boolean STATE variable to transition from an active (inactive) into an inactive (active) state with probability per unit time $p_t = k_{off}\, STATE$ ($p_t = k_{on}(1-STATE)$). Production can occur with propensity: $p_t = \begin{cases} p_{on} & \text{if } STATE = 1 \\ 0 & \text{if } STATE = 0 \end{cases}$. The production probability per unit time in the ON state p_{on} is related to the burst size $bs = \frac{p_{on}}{k_{off}}$, where bs is defined as the mean number of transcripts produced per ON-OFF cycle. In order to be considered bursty transcription, two conditions have to be met (Dar et al., 2012; Shahrezaei and Swain, 2008): $k_{off} \gg k_{on}$ with a burst size $bs \gg 1$. To investigate whether *FLC* production could be bursty, we set the off rate sufficiently fast, $k_{off} = 0.1\ s^{-1}$, which effectively ensured that the first condition was met (see below). The total production rate (per locus) has to be equal to the time-averaged transcription rate (per locus), leading to: $F(V) = \frac{\beta V d}{N_{loci}} = \frac{p_{on}}{k_{off}} \frac{1}{1+\frac{k_{on}}{k_{off}}} k_{on} = bs \frac{1}{1+\frac{k_{on}}{k_{off}}} k_{on}$. Here N_{loci} represents the number of *FLC* loci in each cell. In the main text we first described a cellular ON/OFF production model: in this case N_{loci} equals 1. We then also simulated the more realistic cases where ON/OFF transcription occurs from $N_{loci} = 2$ or 4 (independent) loci. These considerations lead to two distinct scenarios:

1) Burst size scales with volume, as proposed for mammalian genes (Padovan-Merhar et al., 2015), with burst frequency k_{on} independent of volume:

$$bs(V) = \langle bs \rangle \frac{V}{\langle V \rangle} \text{ and } k_{on} = \frac{k_{off} \beta \langle V \rangle d}{k_{off} N_{loci} \langle bs \rangle - \beta \langle V \rangle d}.$$

Here $\langle bs \rangle$ is a chosen constant, the burst size in a cell of average volume $\langle V \rangle = 1.8 pL$.

2) Burst size bs is a chosen constant and burst frequency scales with volume: $k_{on} = \frac{k_{off} \beta V d}{k_{off} N_{loci} bs - \beta V d}$.

To investigate how large the burst sizes could be, bs (and in scenario 2: $\langle bs \rangle$) was then systematically varied from 1 upwards. Through the expressions above for each scenario, setting these burst size parameters then fully determined both the burst frequency k_{on} and p_{on}.

As a model input parameter, we provided to each simulation an experimentally observed cell volume V (Figure S1F). To obtain robust model distributions of the simulated mRNA levels, we repeated the procedures described above over 50 simulations for each cellular volume as observed from the full-length *FLC* intron 1 data set (Figure S1F). We then repeated this procedure for N_{loci} batches, resulting in 10000 simulations per batch. Simulations started at time $t = 0$ and ran until (simulated) time t, updated according to the Gillespie algorithm, exceeded a predefined time of 10 days, to allow the system to reach steady state. We then output the cellular volume and simulation *FLC* mRNA levels (Figure S1G). For the simulations with $N_{loci} = 2$ and 4, we summed the 10000 simulations from the first batch with the other batches to generate 10000 simulated cells with associated cellular mRNA levels, in accordance with the observed cellular volume distribution. We then compared the experimentally observed cellular mRNA distribution (n = 209, Figure 2C) with the 10000 simulated cells using a Levene's test, a Brown-Forsythe test and a Kolmogorov-Smirnov test. The former two test for equal variance by using the mean or median in their test statistics, while the latter tests for equal distributions. When the resulting p values were smaller than 0.05, we considered the chosen burst size incompatible with our experimental results. Maximal consistent burst sizes were, respectively:

Scenario 1, burst size scales with volume:
Levene's test: 3 (N_{loci}=1: p=0.10, N_{loci}=2: p=0.07, N_{loci}=4: p=0.05);
Brown-Forsythe test: 5 (N_{loci}=1: p=0.07, N_{loci}=2: p=0.05) and 6 (N_{loci}=4: p=0.05);
Kolmogorov-Smirnov test: 8 (N_{loci}=1: p=0.05) and 7 (N_{loci}=2: p=0.07, N_{loci}=4: p=0.06).
Scenario 2, burst frequency scales with volume:
Levene's test: 3 (N_{loci}=1: p=0.05, N_{loci}=2: p=0.06, N_{loci}=4: p=0.06);
Brown-Forsythe test: 4 (N_{loci}=1: p=0.06, N_{loci}=2: p=0.09, N_{loci}=4: p=0.06);
Kolmogorov-Smirnov test: 5 (N_{loci}=1: p=0.06, N_{loci}=2: p=0.07, N_{loci}=4: p=0.08).

Stochastic Simulations of Spatiotemporal *FLC* Transcriptional Dynamics

To quantify mRNA release from the *FLC* locus (Figure 3A), we simulated stochastic *FLC* transcription and RNA dynamics in root outer layer cells by implementing a spatiotemporal Gillespie algorithm (Slepoy et al., 2008) in C++ (https://github.com/ri23/FISHmodel). The reactions included are described below and illustrated in Figure S3A.

The *FLC* locus ($\sim$ 6 kb) was divided into L=209 sites of length N_{bp}= 30 bp (see Figure S3A for a graphical representation). The sites are numbered 0...(L-1). The sense / antisense Transcription Start Sites (TSSs) are represented by site 4 and 208 respectively. Each site can be occupied by at most one Pol II. If a TSS site is unoccupied, a Pol II can bind to that TSS. We refer to this process as transcription initiation. Since outer layer cells exhibit very low antisense expression (Figures 4B and 4C), we set the antisense transcription initiation rate to zero. Once a Pol II has bound to a sense TSS we assume it is competent to elongate in the sense direction. Of course, transcription initiation, subsequent formation of a transcription elongation complex and possibly promoter proximal pausing are themselves complex processes. Nevertheless, within our minimal modelling approach we account for these processes within a single "coarse-grained" transcriptional initiation probability. Incorporating these processes in more detail would not qualitatively alter our conclusions on *FLC* regulation. If the TSS site is unoccupied, sense initiation can occur with probability per unit time (propensity): $p_t = \frac{\beta V d}{N_{loci}}$.

Based on previously published Pol ChIP expression in ColFRI (Li et al., 2015) and consistent with previous modelling of *FLC* regulation (Wu et al., 2016), we assume the magnitude of the elongation rate is independent of position along the *FLC* gene. Furthermore, within our minimal modelling approach we do not explicitly incorporate Pol II pausing, backtracking or arrest. Including these processes in more detail would not qualitatively alter our conclusions. In case of sense transcription, a Pol II at site i (P_i = 1) can elongate to a neighbouring site i+1, if that neighbouring site is unoccupied, with propensity: $p_t = \frac{v}{N_{bp}} P_i$

Here, v = 3 bp/s represents the elongation rate, consistent with the range determined through our parameter inference procedure (Figure 3D).

Consistent with previous findings (Wu et al., 2016), we assume that there is no early termination, only termination of a transcribing Pol II as a consequence of cleavage/polyadenylation of the RNA transcript. We used the annotated RNA 3′ ends to determine where Pol II could drop off the template after elongation. We assume that Pol II ceases to elongate soon after it transcribes its canonical pA sequence (Wu et al., 2016): when a sense Pol II reaches the Transcription End Site (TES), site 204, it can terminate with probability per unit time of $k_{pA} = 0.02 s^{-1}$, resulting in a free Pol II and a 3′ processed sense transcript that remains at the locus (Figure S3A). The exact value of k_{pA} has little influence on our results as long it is shorter than the mRNA release rate $k_{x,}$ as is the case in our choice of parameters (see below).

The creation of sense RNA is modelled as follows (Figure S3A). A sense Pol II at site i (P_i) has produced unspliced RNA corresponding to the sites TSS…i-1. Splicing of sense *FLC* intron 1 is explicitly modelled: as soon as Pol II elongates past the intron 1 acceptor site I1A=131, Pol II can continue to elongate and, in addition, splicing of intron 1 can occur with a probability per unit time $\sigma = 1.5 \times 10^{-3} s^{-1}$, consistent with the range determined through our parameter inference method (Figure 3D). This reaction results in a Pol II with nascent, spliced RNA attached: P_i^s. This Pol II species can elongate with the same dynamics as for Pol II with unspliced nascent RNA (P_i). After splicing, lariat degradation is assumed here to occur immediately (see below). In the main text, the σ^{-1} timescale was defined slightly differently as the time from when intron 1 is completely transcribed to the moment of splicing, plus the extra waiting time until lariat degradation begins. However, our results (Figure 3) do not depend on this model simplification because our simulation output, i.e. all the RNA species that were measured experimentally (described below in detail), are unaffected by this detail. Therefore, despite this simplification, we can directly compare the simulation output with our experimental observations.

A splicing reaction results in cleaved intronic (lariat) RNA with the 5′ end at site I1D=14 and 3′ end at site I1A-1. We term this RNA species $IN_{I1D,I1A-1}$, with the first index indicating the 5′ end and the second index the 3′ end. This RNA and in general $IN_{i,j}$ can then be degraded from 5′ to 3′ in a first order reaction with rate k_{53} and propensity $p_t = \frac{k_{53}}{N_{bp}} IN_{i,j}$. As a result of this reaction, $IN_{i+1,j}$ is formed, corresponding to intronic RNA with a 5′ end at site i+1 (and 3′ end at j>i+1). In the case of the last step of intron RNA degradation such that i+1=j, this reaction occurs without a reaction product. Similar to 5′ to 3′ degradation, we also allowed 3′ to 5′ lariat degradation with propensity $p_t = \frac{k_{35}}{N_{bp}} IN_{i,j}$ resulting in reaction product, $IN_{i,j-1}$. The k_{53} and k_{35} estimates were both set to 2 bp/s consistent with our experimental estimates.

We only explicitly modelled splicing reactions of sense intron 1 in the simulations. Incorporating splicing of additional sense introns in the model would not affect our results provided that each splicing reaction is independent of the others. Splicing can also occur after sense Pol II has terminated (Figure S3A). If Pol II has terminated with intron 1 spliced out, *s1FLC* is created, a cleaved full-length RNA at the locus with intron 1 spliced out. If Pol II has terminated prior to splicing of intron 1, a full-length, unspliced RNA termed *unsFLC* is produced. *unsFLC* can be spliced in intron 1 with probability per unit time σ resulting in *s1FLC* and $IN_{I1D,I1A-1}$. The lariat can then be degraded as described above. *s1FLC* can be released from the locus with probability per unit time: $p_t = k_x s1FLC$, i.e. we performed simulations with a constant export rate $k_x = 5 \times 10^{-4} s^{-1}$ in the presence of missing length fraction f_{Lp}=*1/3*, see below. Lastly, *FLC* mRNA release from the locus leads to a mature (spliced) sense *FLC* mRNA: *sFLC*. This mature transcript can then be degraded with probability per unit time d.

We output the cell volume, simulation RNA levels corresponding to *FLC* mRNA, the number of RNAs corresponding to the four intron 1 probe sets (I_f,$I_{5'}$ I_m and $I_{3'}$) and the number of exonic RNAs (R_{loc}, only considered in further analyses when $I_f > 0$), whilst taking into consideration a missing length fraction f_{Lp}=1/3, as follows:

$$I_f = unsFLC + \sum_{i=I1D+\frac{2}{3}(I1A-I1D)}^{L-1} P_i + \sum_{i=I1D}^{I1D+\frac{1}{3}(I1A-I1D)-1} \sum_{j=i+\frac{2}{3}(I1A-I1D)}^{I1A-1} IN_{i,j};$$

$$I_m = unsFLC + \sum_{i=I1D+\frac{7}{12}(I1A-I1D)}^{L-1} P_i + \sum_{i=I1D}^{I1D+\frac{1}{4}(I1A-I1D)-1} \sum_{j=I1D+\frac{7}{12}(I1A-I1D)}^{I1A-1} IN_{i,j} + \sum_{i=I1D+\frac{1}{4}(I1A-I1D)}^{I1D+\frac{5}{12}(I1A-I1D)-1} \sum_{j=i+\frac{1}{3}(I1A-I1D)}^{I1A-1} IN_{i,j};$$

$$I_{5'} = unsFLC + \sum_{i=I1D+\frac{1}{3}(I1A-I1D)}^{L-1} P_i + \sum_{i=I1D}^{I1D+\frac{1}{6}(I1A-I1D)-1} \left(\sum_{j=i+\frac{1}{3}(I1A-I1D)}^{I1D+\frac{1}{2}(I1A-I1D)-1} IN_{i,j} + \sum_{j=I1D+\frac{1}{2}(I1A-I1D)}^{I1A-1} IN_{i,j} \right);$$

$$I_{3'} = unsFLC + \sum_{i=I1D+\frac{5}{6}(I1A-I1D)}^{L-1} P_i + \sum_{i=I1D}^{I1D+\frac{1}{2}(I1A-I1D)-1} \sum_{j=I1D+\frac{5}{6}(I1A-I1D)}^{I1A-1} IN_{i,j} + \sum_{i=I1D+\frac{1}{2}(I1A-I1D)}^{I1D+\frac{2}{3}(I1A-I1D)-1} \sum_{j=i+\frac{1}{3}(I1A-I1D)}^{I1A-1} IN_{i,j};$$

$$R_{loc} = unsFLC + s1FLC + \sum_{i=sTES-11}^{sTES} \left(P_i + P_i^s\right).$$

The number of cellular *FLC* mRNA equals: $N_{loci} \times sFLC$. The number of intron foci per cell were then for each simulation calculated as $S_j = N_{loci} I_J$ with $j \in \{f, 5', m, 3'\}$. Simulated time and number of simulations were as described for the cellular *FLC* mRNA dynamics simulations. Calculations of population average and averages for various attained volumes were performed in the same manner as described above for the experimental data points.

Quantification of *FLC* mRNA Release from the Locus

To determine the amount of exonic *FLC* RNA at the locus given the presence of *FLC* full-length intron 1, we utilized FISH-quant, a MATLAB software suite (Mueller et al., 2013). We followed the quantification procedures as detailed in the manual available from http://dev.mri.cnrs.fr/documents/95 . This method requires abundant mRNA smFISH signal as well as a method to indicate the locations of loci: in our case *FLC* full-length intron 1 smFISH signal as this co-localizes exclusively with *FLC* loci (Figure S1A). Cell area outlines (using an overlay of mRNA and DAPI signal) and loci (using the intron 1 focus signal) were segmented manually. FISH-quant then determined computationally for each cell the (predominantly cytoplasmic) mRNA foci locations and background intensity profile. The resulting foci were manually inspected to confirm the accuracy of the algorithm. Cell areas and corresponding mRNA counts were then output to a text file. Cell areas were then converted into cell volumes using the projection method described above. As part of the mature mRNA quantification procedure, an overall average mRNA spatial intensity focus profile (point spread function, PSF) was then calculated from all determined mRNA foci. This background corrected intensity profile represents one mRNA molecule. The next step was to quantitatively compare the mRNA signal at the indicated loci with the PSF. Here, we considered three different algorithms that have been shown to be accurate for intensity quantification in the regime of relatively low RNA copy number with a spatially confined transcription site (Mueller et al., 2013):

1. Comparison of integrated intensity of the transcription site and individual mRNA molecules (Int).
2. Superimposition of PSFs to reconstruct an image of the transcription site (PSF).
3. Comparison of the estimated amplitude of the transcription site and individual mRNA molecules (Amp).

The results which indicated the number of molecules for each transcription site were then output. Lastly, we manually included the appropriate cell area as obtained from the mature mRNA output in the nascent RNA output list in order to relate cell area and exonic RNA number. The resulting three distributions were relatively similar, albeit with means varying from 1.9 up to 2.5 exonic RNA molecules (Figure 3H).

DATA AND SOFTWARE AVAILABILITY

Cell volume and area estimation and RNA foci count code, as well as code for stochastic simulations of (1) *FLC* mRNA production and degradation and (2) *FLC* transcription and RNA processing kinetics, all as described above, are available on https://github.com/ri23/FISHmodel. FISH-quant software, used to estimate the number of exonic *FLC* RNA at the locus, is available on https://code.google.com/archive/p/fish-quant/.

Cell Systems, Volume *4*

Supplemental Information

Cell-Size-Dependent Transcription of *FLC* and Its Antisense Long Non-coding RNA *COOLAIR* Explain Cell-to-Cell Expression Variation

Robert Ietswaart, Stefanie Rosa, Zhe Wu, Caroline Dean, and Martin Howard

Supplemental Information

Figure legends

Figure S1. Related to Figure 2

Characterization of cellular *FLC* expression, mRNA degradation variation and cellular volume

(A) Fluorescence localization of *FLC* DNA as assayed by DNA-FISH, full length *FLC* intron 1 RNA as assayed by smFISH (red), and an overlay containing both the above and DAPI stain (blue) in representative ColFRI outer layer root cells. Scale bar: 5µm. Data previously published in (Rosa et al., 2016).

(B) Histogram of pooled cellular *FLC* mRNA distributions after treatment with DMSO (mock) for 4 h and 6 h (N=202 cells from 3 biological replicates).

(C) Histogram of cellular *FLC* mRNA counts after 6 h of ActD treatment (blue) and Poisson decay process prediction (red) with distribution of mRNA after 4 h ActD treatment (shown in Fig. 2E) as the initial distribution. Right panel: Cumulative distribution functions (CDFs) of the *FLC* mRNA count and model prediction.

(D) Scatter plot comparing two volume estimation methods: 3D segmentation vs projection method in ColFRI outer layer root cells from where *FLC* mRNA counts were recorded (N=209 cells from 8 biological replicates, Fig. 2C). Also shown is line with unit slope (black).

(**E**) Schematic of reactions implemented in stochastic Gillespie simulations of cellular *FLC* mRNA production, with either Poisson or ON/OFF dynamics, and degradation.

(F) Histogram of cell volume distribution (N=200 cells from 3 biological replicates) from root outer layer cells from where full length intron 1 foci counts were recorded (Fig. 3B,C). These cell volumes were used as an input into the stochastic simulations described in (E) to account for the variation in cell size affecting *FLC* transcription.

(**G**) Histograms of cellular mRNA model predictions from stochastic simulations described in (E) given the experimentally observed volume distribution described in (F). ON/OFF simulations had a burst size of 3 transcription events per ON/OFF cycle with either the burst frequency (k_{on}) or burst size (*bs*) scaling with cell volume. Right panel: cumulative distribution functions (CDFs) of *FLC* mRNA (from Fig. 2C) and model predictions.

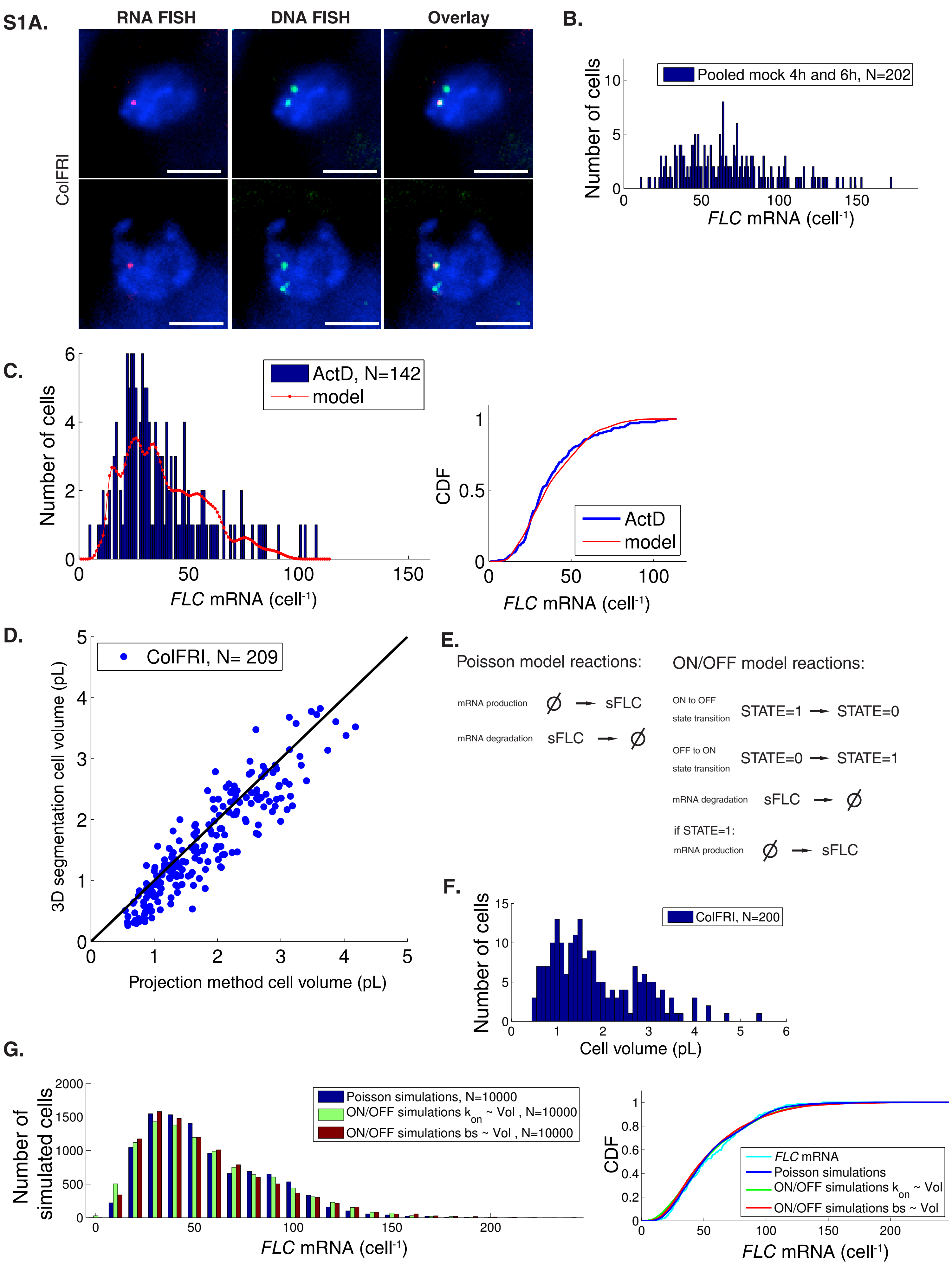

S1A.
RNA FISH
DNA FISH
Overlay
ColFRI
B.
Pooled mock 4h and 6h, N=202
Number of cells
FLC mRNA (cell-1)
C.
ActD, N=142
model
Number of cells
FLC mRNA (cell-1)
CDF
ActD
model
FLC mRNA (cell-1)
D.
ColFRI, N= 209
3D segmentation cell volume (pL)
Projection method cell volume (pL)
E.
Poisson model reactions:
mRNA production ∅ → sFLC
mRNA degradation sFLC → ∅
ON/OFF model reactions:
ON to OFF state transition STATE=1 → STATE=0
OFF to ON state transition STATE=0 → STATE=1
mRNA degradation sFLC → ∅
if STATE=1: mRNA production ∅ → sFLC
F.
ColFRI, N=200
Number of cells
Cell volume (pL)
G.
Poisson simulations, N=10000
ON/OFF simulations kon ~ Vol , N=10000
ON/OFF simulations bs ~ Vol , N=10000
Number of simulated cells
FLC mRNA (cell-1)
CDF
FLC mRNA
Poisson simulations
ON/OFF simulations kon ~ Vol
ON/OFF simulations bs ~ Vol
FLC mRNA (cell-1)

Figure S2. Related to Figure 3

Estimation of Pol II elongation, *FLC* intron 1 processing and lariat degradation rates using a range of missing probe length fractions

(**A**) Marginal distributions for intron processing rate that generate good fits to the data shown in Fig. 3C according to a χ^2 test (degrees of freedom k=4, acceptance probability $p \geq 0.1$) with missing probe length fractions ranging from 0 to 1/3. Boxplots indicate: minimum, 25% quantile, median, 75% quantile and maximal values.

(**B**) As in (**A**) but for elongation rate.

(**C**) As in (**A**) but for 5' to 3' lariat degradation rate.

(**D**) As in (**A**) but for 3' to 5' lariat degradation rate.

(**E**) Volume dependence of replicates measuring cellular smFISH foci counts per cell in outer layer cells for four different *FLC* intron 1 probe sets: full length (full), middle (mid), 5' end and 3' end. Full length: two separate experiments were performed using respectively 4 and 3 biological replicates, respectively. Experiment 1: data as in Fig. 2B and 4C, experiment 2: data as in Fig. 3C. For the other probe sets (mid, 5' end and 3' end) replicates are shown as pooled data from two biological replicates each; for these, all biological replicates pooled together are shown in Fig. 3E,F. Error lines: s.e.m. as function of volume.

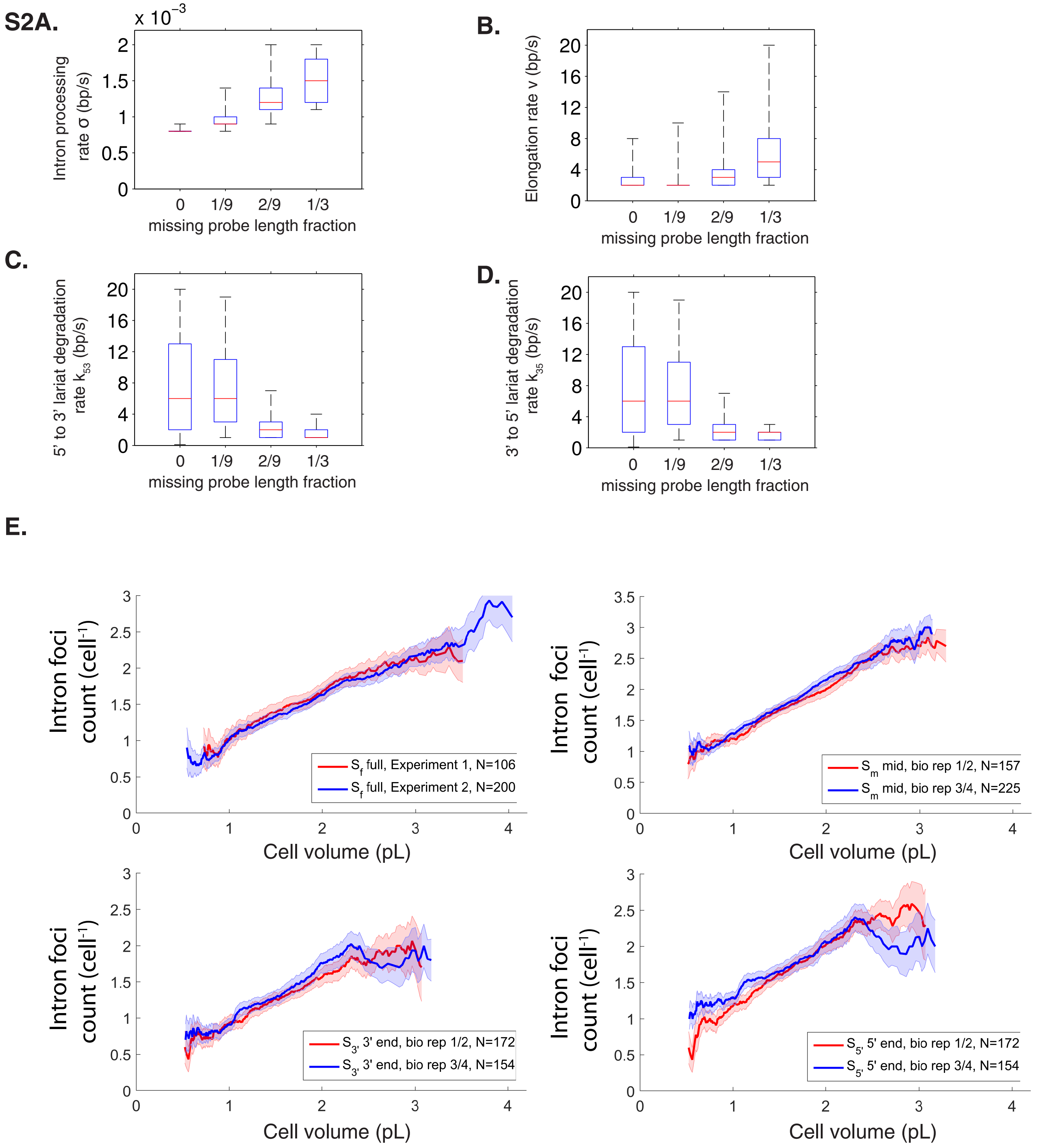
S2A.
x 10^-3
Intron processing rate σ (bp/s)
missing probe length fraction
0
1/9
2/9
1/3
B.
Elongation rate v (bp/s)
missing probe length fraction
C.
5' to 3' lariat degradation rate k53 (bp/s)
missing probe length fraction
D.
3' to 5' lariat degradation rate k35 (bp/s)
missing probe length fraction
E.
Intron foci count (cell-1)
Cell volume (pL)
Sf full, Experiment 1, N=106
Sf full, Experiment 2, N=200
Sm mid, bio rep 1/2, N=157
Sm mid, bio rep 3/4, N=225
S3' 3' end, bio rep 1/2, N=172
S3' 3' end, bio rep 3/4, N=154
S5' 5' end, bio rep 1/2, N=172
S5' 5' end, bio rep 3/4, N=154

Figure S3. Related to Figure 3

Spatiotemporal Gillespie simulations of *FLC* transcription to quantify *FLC* mRNA release from the locus

(A) Schematic of spatially discretized *FLC* gene and reactions as implemented in spatiotemporal stochastic Gillespie simulations of *FLC* transcription and RNA degradation dynamics. Sense (black) and antisense (grey) transcripts are indicated. Since antisense transcription is very low in root outer layer cells, we did not include antisense transcription in our simulations.

(B) Volume dependence of cellular smFISH foci counts per cell in outer layer cells for *FLC* intron 1 as in Fig 3E,F. In left panel: full length (full) and 5' end; in right panel: middle (mid) and 3' end. Also shown are spatiotemporal Gillespie simulation fits (black, grey). Error lines: s.e.m. as function of volume.

(**C**) Volume dependence of average locus associated exonic *FLC* RNA levels from two separate experiments (with 2 biological replicates per experiment). Three different quantification methods shown as in Fig. 3H, which shows the pooled data. Error lines: s.e.m. as a function of volume.

S3A.

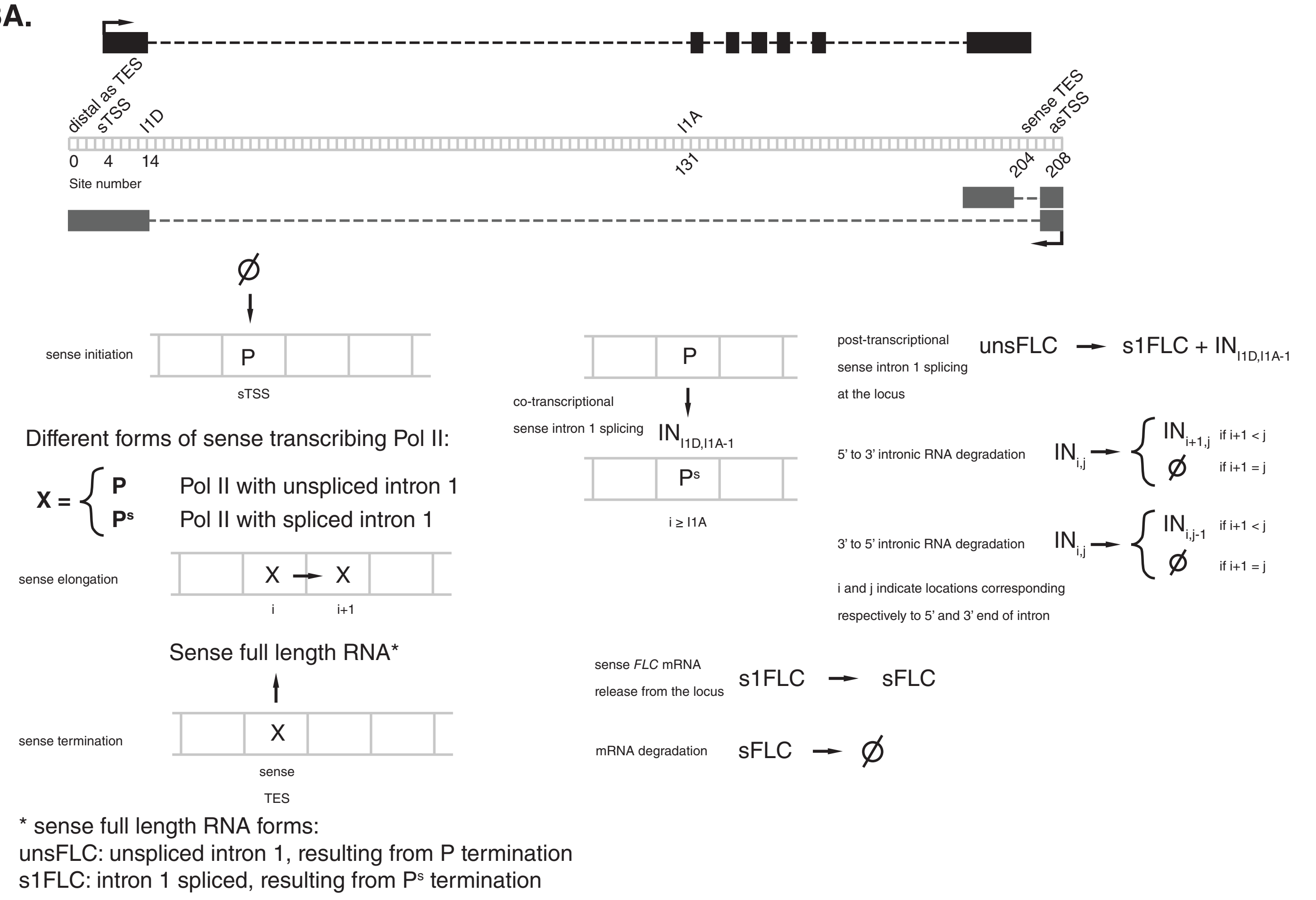
distal as TES
sTSS
I1D
I1A
sense TES
asTSS
0
4
14
131
204
208
Site number
sense initiation
P
sTSS
Different forms of sense transcribing Pol II:
X =
P
Pol II with unspliced intron 1
Ps
Pol II with spliced intron 1
sense elongation
X → X
i
i+1
Sense full length RNA*
sense termination
X
sense
TES
* sense full length RNA forms:
unsFLC: unspliced intron 1, resulting from P termination
s1FLC: intron 1 spliced, resulting from Ps termination
co-transcriptional
sense intron 1 splicing
IN I1D,I1A-1
i ≥ I1A
post-transcriptional
sense intron 1 splicing
at the locus
unsFLC → s1FLC + IN I1D,I1A-1
5' to 3' intronic RNA degradation
IN i,j
IN i+1,j
if i+1 < j
if i+1 = j
3' to 5' intronic RNA degradation
IN i,j-1
if i+1 < j
if i+1 = j
i and j indicate locations corresponding
respectively to 5' and 3' end of intron
sense FLC mRNA
release from the locus
s1FLC → sFLC
mRNA degradation
sFLC → Ø

B.

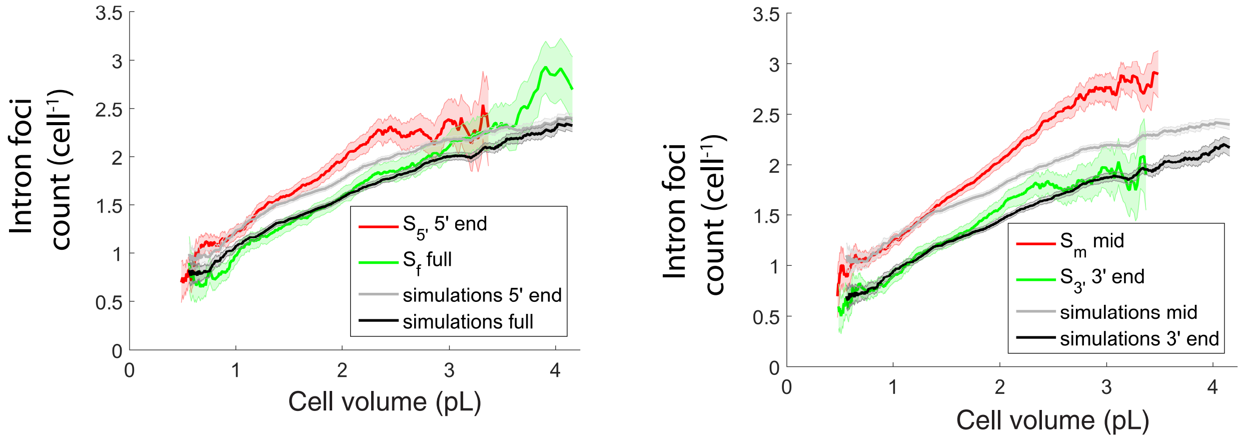
Intron foci
count (cell-1)
Cell volume (pL)
S5', 5' end
Sf full
simulations 5' end
simulations full
Sm mid
S3', 3' end
simulations mid
simulations 3' end

C.

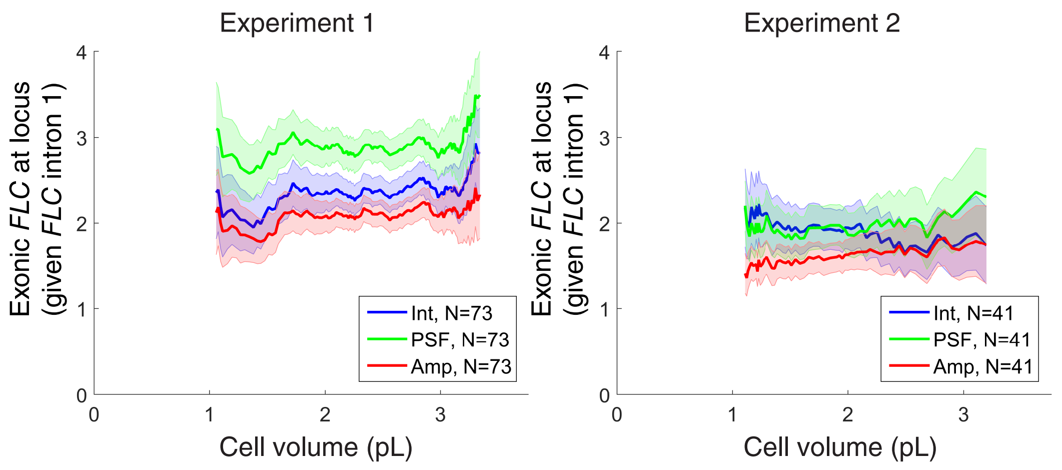
Experiment 1
Experiment 2
Exonic FLC at locus
(given FLC intron 1)
Cell volume (pL)
Int, N=73
PSF, N=73
Amp, N=73
Int, N=41
PSF, N=41
Amp, N=41

Figure S4. Related to Figure 4.

Characterization of anticorrelation between *FLC* sense and antisense transcription

(A) Fluorescence localization of full length sense intron 1 (red) and antisense (*COOLAIR*) 5' end distal intron (green) overlaid with DAPI stain (blue) in representative ColFRI prevasculature root cell. Scale bar: 5µm. Data previously published in (Rosa et al., 2016).

(B) Frequency of loci containing *FLC* full length intron 1, when *COOLAIR* 5' end distal intron is not present (S) or present (S if AS) at the same locus in root prevasculature cells. N=254 cells from 6 biological replicates. Error bars: s.e.m. **** p-value < 10^{-4}. Data previously published in (Rosa et al., 2016).

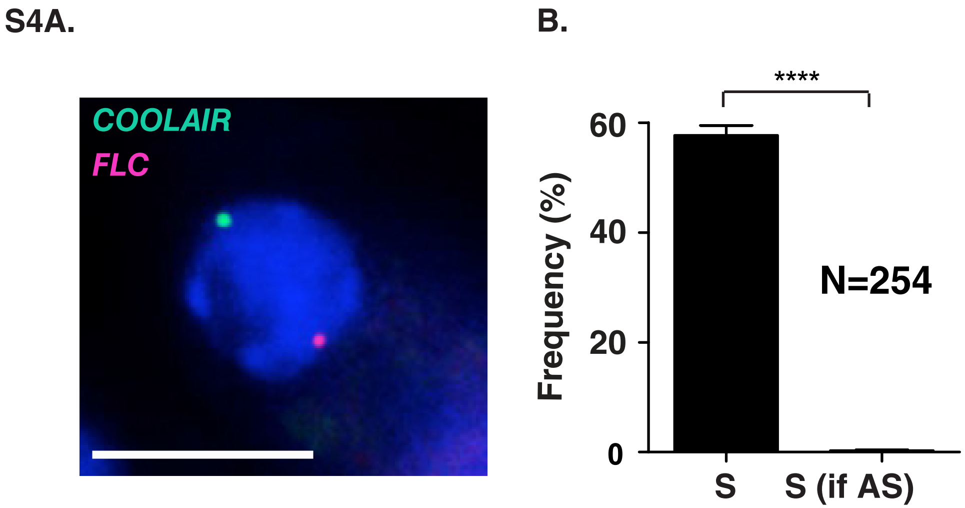
S4A.
COOLAIR
FLC
B.
****
60
40
20
0
Frequency (%)
N=254
S
S (if AS)
